# Noble metal-free CZTS electrocatalysis: synergetic characteristics and emerging applications towards water splitting reactions

**DOI:** 10.3389/fchem.2024.1394191

**Published:** 2024-05-30

**Authors:** Somnath C. Dhawale, Renuka V. Digraskar, Anil V. Ghule, Bhaskar R. Sathe

**Affiliations:** ^1^ Department of Chemistry, Dr. Babasaheb Ambedkar Marathwada University, Chhatrapati Sambhajinagar, Maharashtra, India; ^2^ Department of Nanotechnology, Dr. Babasaheb Ambedkar Marathwada University, Chhatrapati Sambhajinagar, Maharashtra, India; ^3^ Department of Chemistry, Savitribai Phule Pune University, Pune, India; ^4^ Department of Chemistry, Shivaji University, Kolhapur, Maharashtra, India

**Keywords:** CZTS nanoparticles, water splitting reactions, hydrogen evolution reactions, oxygen evolution reactions, Ni, CO and Fe-doped CZTS, amine functionalized CZTS, MoS_2_-CZTS

## Abstract

This review provides a comprehensive overview of the production and modification of CZTS nanoparticles (NPs) and their application in electrocatalysis for water splitting. Various aspects, including surface modification, heterostructure design with carbon nanostructured materials, and tunable electrocatalytic studies, are discussed. A key focus is the synthesis of small CZTS nanoparticles with tunable reactivity, emphasizing the sonochemical method’s role in their formation. Despite CZTS’s affordability, it often exhibits poor hydrogen evolution reaction (HER) behavior. Carbon materials like graphene, carbon nanotubes, and C_60_ are highlighted for their ability to enhance electrocatalytic activity due to their unique properties. The review also discusses the amine functionalization of graphene oxide/CZTS composites, which enhances overall water splitting performance. Doping with non-noble metals such as Fe, Co., and Ni is presented as an effective strategy to improve catalytic activity. Additionally, the synthesis of heterostructures consisting of CZTS nanoparticles attached to MoS_2_-reduced graphene oxide (rGO) hybrids is explored, showing enhanced HER activity compared to pure CZTS and MoS_2_. The growing demand for energy and the need for efficient renewable energy sources, particularly hydrogen generation, are driving research in this field. The review aims to demonstrate the potential of CZTS-based electrocatalysts for high-performance and cost-effective hydrogen generation with low environmental impact. Vacuum-based and non-vacuum-based methods for fabricating CZTS are discussed, with a focus on simplicity and efficiency. Future developments in CZTS-based electrocatalysts include enhancing activity and stability, improving charge transfer mechanisms, ensuring cost-effectiveness and scalability, increasing durability, integrating with renewable energy sources, and gaining deeper insight into reaction processes. Overall, CZTS-based electrocatalysts show great promise for sustainable hydrogen generation, with ongoing research focused on improving performance and advancing their practical applications.

## 1 Introduction: energy and electrochemical reactions

The most challenging topic at the moment is the rising worldwide demand for energy, the discussion surrounding its scarcity, and its associated environmental effects. The COVID-19 shutdown in 2020 harmed the economy, although, over the previous 2 decades, the use of renewable energy sources like wind and photovoltaic solar cells has increased significantly. The urgency of the new energy economy has been accelerated by political action, technological advancement, and the mounting need to combat climate change. Even if the switch to this new energy economy will be quick and simple, there is no assurance that it will be finished in time to prevent severe weather catastrophes. The future energy economy, however, is poised to be significantly different from our current model (Energy and Agency, 2021). However, traditional fossil fuel depletion has exacerbated social and environmental problems. Nowadays, researchers are being pushed to identify renewable and environmentally acceptable energy sources as a result of the growing global energy crisis and associated environmental concerns. Therefore, hydrogen (H_2_) energy is the most practical and environmentally friendly energy source (Wang et al., 2020). Photoelectric water splitting with semiconductor nanoparticles is the most efficient hydrogen generation strategy. On the other hand, the inherent toxicity of most photocatalysts, as well as the wide optical band gap, have impeded the development of practical, full, and long-term solar-powered hydrogen production systems. As a result, developing high-efficiency, non-toxic photocatalysts with a large optical absorption zone continues to be difficult. The goal of practical solar hydrogen evolution applications is to create photocathodes that are high-efficiency, low-cost, and environmentally benign, as well as stably stable throughout time (Huang et al., 2021). The kinetics of electrocatalytic reaction processes, such as the oxygen reduction reaction (ORR), oxygen evolution reaction (OER), and hydrogen evolution reaction (HER), are significantly influenced by the kinetics of advanced devices involving electrochemical processes for clean energy conversion (Zhang et al., 2014; Zhang et al., 2014; Lee et al., 2014; O’Heir, 2017). Despite their differences in mechanisms in homogeneous and heterogeneous electrocatalyst, most heterogeneous electrochemical processes have some common features such as occurring at the solid-liquid interface and involving multistep ion/electron linked electron transfer reactions, which results in extremely slow electron transfer kinetics and necessitates the use of highly efficient electrocatalysts for water splitting reactions.

The water-splitting systems that use an alkaline solution as an electrolyte, neutral and acidic media also provide major advantages. Acidic proton exchange membrane (PEM) electrolyzers, for example, are more compact, have superior gas purity and voltage efficiency, and have a neutral working environment—which is better suited for mass production due to its low cost, inherent benign nature, and weak causticity. Therefore, the development of extremely effective noble-metal or metal-free electrocatalysts is crucial for overall water splitting in acidic or neutral electrolytes (Yu et al., 2021). A polycrystalline semiconductor material with photoelectric properties is called CZTS. Because of its structural identity, this substance is commonly referred to as kesterite material. This chemical was selected for thin-film solar cells due to its availability, non-toxicity, and environmental friendliness. When compared to the CIGS material, which contains tellurium, indium, and cadmium at 0.16 ppm, 0.15 ppm, and 0.001 ppm, respectively, the abundance of the CZTS materials constituent elements—copper, zinc, tin, and sulfur on earth is 68, 79, 2.2, and 420 ppm, respectively. The material’s absorption of light is attributed to CZTS, a quaternary compound with a kesterite structure similar to CIGS. The CZTS material is stable over time due to its kesterite structure. When exposed to light, CZTS exhibits a highly noticeable hole-electron pair production rate and a greater absorption coefficient (105 cm^−1^) (Chirilă et al., 2013). The material is ideal for the absorber layer since it possesses a straight optical band gap. With respect to the various preparation techniques and doping/replacement of the CZTS material, it exhibits an adjustable band gap ranging from 1.4 to 1.6 eV. The substance functions as an organic p-type semiconductor. According to recent studies, selenium (Se) can completely replace sulfur (S) to lower the band gap of the former to 1 eV. The material’s inherent qualities make it a very promising candidate for use as an absorber layer in thin-film solar cells (Ge et al., 2015).

The economy and environment can be greatly affected by a multifunctional semiconductor nanoparticle (NP) that can be used in more than two ways. Due to its excellent direct band gap (1.4–1.6 eV) and high absorption coefficient, quaternary multivalent Cu_2_ZnSnS_4_ has been thoroughly researched for use in solar systems (Suryawanshi et al., 2017). Nevertheless, quaternary chalcogenides have lately attracted attention for their potential in thermoelectric and photocatalytic applications, demonstrating its multifunctionality, in addition to solar energy harvesting. Advances in synthetic techniques have made it feasible to address the difficulty of preserving the homogeneity, stoichiometry, and repeatability of these complex quaternary compounds. Moreover, the development of sophisticated techniques for the synthesis of surfactant-free nanomaterials may facilitate the implementation of important yet affordable applications. The adjustable shapes, low toxicity, and abundance of elements in quaternary copper-based chalcogenides, including Cu−Zn−Sn−S nanocrystals (CZTS NCs), have made them attractive nanomaterials for photocatalysis and electrocatalysis (Mensah-Darkwa et al., 2022). The development of CZTS nanomaterials for hydrogen evolution processes (HERs) with a variety of sizes, shapes, and crystalline phases has been the focus of recent efforts. The literature reports on a variety of synthesis techniques, including as sonochemical, colloidal, and solvothermal techniques. Nevertheless, a lot of these techniques call for high pressure and temperature, which produces multi-sized and polydisperse nanocrystals. Surface ligands are critical to the colloidal method’s ability to produce high-quality, well-monodispersed nanocrystals. The growth phase of nanocrystals can be influenced by the morphologies and dimensions of individual crystal faces via the selective adsorption of ligands (Li et al., 2014; Digraskar et al., 2017).

The three types of tetragonal crystal structures that CZTS might potentially form are stannite, kesterite, and primitive-mixed CuAu (PMCA) structures. By replacing the in atoms with Zn and Sn, the chalcopyrite-type CuInS_2_ (CIS) structure is converted to a kesterite-type structure. The layers of kesterite formations alternate at Z = 0, 1/2, 1/2, and ¾ in the following order: CuSn, CuZn, CuZn, and CuZn. The anions and cations in the kesterite CZTS crystal are organized in a tetrahedral bonding arrangement, with a stacking mechanism that is similar to zinc mixes like ZnO or ZnS. The ZnSn layer in the stannite structure alternates with the Cu_2_ layer, whereas the layer alignment of the kesterite structure is addressed above. This is the only difference between the stannite and kesterite structures. PMCA is a unique structure consisting of two kesterite structures and a stannite unit cell (Mitzi et al., 2011). The kesterite structure has more thermodynamic stability than the other two of the three potential structures of CZTS. It has so been selected above other constructions. According to both theory and experiment, tetragonal kesterite possesses the most stable crystal structure for Cu_2_ZnSn(S, Se)_4_ (Persson, 2010). However, a variety of additional metastable forms, including wurtzite, stannite, zinc blende, and wurtzite-kesterite, might also be found.

The regulated synthesis of nanocrystals is well-known to rely heavily on oleylamine (OM), a common reducing surfactant and solvent. However, more research is needed to determine the underlying modulation process. Therefore, to clarify the crucial function that OM plays in the shape, size, and crystallization of CZTS nanocrystals, systematic studies must be designed. Cu_2_ZnSnS_4_ has other applications outside thermoelectric and photovoltaics, including electrocatalytic water splitting for H_2_/O_2_ evolution and photocatalytic redox processes, then it might be a low-cost multifunctional material with great potential (Zou et al., 2013). Despite its high electroactivity and effectiveness as a catalyst for the hydrogen evolution at low overpotentials, platinum is not appropriate for catalysis on a large enough scale to meet global energy demands due to its expensive cost and limited availability. Because they have lower overpotentials, a number of alternative metal chalcogenides or oxides have been introduced recently to replace expensive metal catalysts. These problems, such as a lack of active sites, inadequate electrical transport, ineffective electrical contact with the catalyst, and instability under operating conditions, have been addressed by chemical exfoliation or functionalization with compounds that offer a high surface area or enhance active sites (Kibsgaard et al., 2014). Nevertheless, the manufacture and research of electrocatalysts are made more difficult by the fact that these methods are frequently severe reaction conditions and are not always synthetically successful. In the photocatalytic water splitting reaction, CZTS, a low-cost photovoltaic material, has demonstrated potential as a p-type semiconductor, effectively creating H_2_ from water. Nevertheless, further research has to be done on its electrochemical behavior in the oxygen evolution reaction (OER) and hydrogen evolution reaction (HER).

An overview of noble-metal-free catalytic materials as complete water-splitting electrocatalysts in neutral or acidic conditions is provided here, focusing on various design approaches to achieve greater water-splitting activities and the underlying activity-structure correlations. An overview of recent developments on noble-metal-free water-splitting catalysts, such as transition-metal oxides, nitrides, carbides, phosphides, chalcogenides, and metal complexes or metal-free carbon under acidic and neutral conditions, follows the introduction of catalytic mechanisms for acidic/neutral water splitting.

## 2 Electrocatalytic water splitting reactions

**FIGURE 1:**
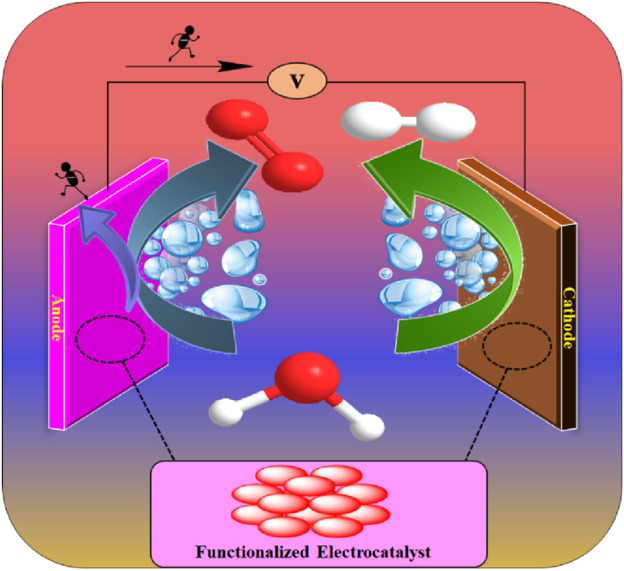
The classic electrolytic cell containing anode, cathode and electrolyte for water splitting reactions.

Electrochemical water splitting is a viable method of generating hydrogen (H_2_), as a clean and sustainable energy carrier and the classic electrolytic cell for water splitting reaction which is shown in Figure 1. The electrocatalytic cell is made up of three portions such as an anode, a cathode which is modified with functionalized electrocatalyst, and an electrolyte. Water molecule can be decomposed into gaseous hydrogen and oxygen on the cathode and anode, respectively with applied external potential to the electrode. The electrochemical water splitting reaction involves two half-cell reactions, such as oxygen evolution reaction (OER) at anode and hydrogen evolution reaction (HER) at cathode, respectively.

Various electrochemical reactions on the electrodes will occur in different electrolytes, even if the overall reaction is the same and associated with splitting of water (Zou and Zhang, 2015; Yan et al., 2016; Joo et al., 2019).

By considering the different electrolytes in which water splitting reactions occurs. In this line, the reactions involved in electrochemical water splitting reactions have been depicted as:

$$\text{Total Reaction}:{2H}_{2}{O\;\to \;2H}_{2}{+O}_{2}$$ (1)

In acidic electrolyte,

$$\text{Cathode}:{2H}^{+}{+2e}^{‐}{\;\to \;H}_{2},\text{Ec}=0\;V$$ (2)

$$\text{Anode}:{2H}_{2}{O\;\to \;O}_{2}{+4H}^{+}{+4e}^{‐},\text{Ea}=1.23\;V$$ (3)

In alkaline electrolyte,

$$\text{Cathode}:{2H}_{2}{O+2e}^{‐}{\;\to \;H}_{2}{+2\text{OH}}^{‐},\text{Ec}=‐0.83\;V$$ (4)

$$\text{Anode}:{4\text{OH}}^{‐}{\;\to \;O}_{2}{+2H}_{2}{O+4e}^{‐}$$ (5)

Under standard temperature and pressure (STP) conditions, the free energy change (∆G^0^) +237.2 KJ per mole of H_2_ is required for one molecule of water converting into hydrogen and oxygen. However, more work is required to expand the gases produced (T∆S^0^), the enthalpy change (∆H^0^ = ∆G^0^ + T∆S^0^) is +286 KJ per mole of H_2_. For water splitting, these values correspond to a reversible electrolysis cell voltage of ∆E^0^_rev_. 298 = 1.23 V vs. RHE and a thermo-neutral (no heat is lost or required) cell voltage of ∆E^0^_th_. 298 = 1.48 V. In an ideal situation if T∆S^0^ is provided externally, just 1.23 V of external potential would be required to initiate water splitting in an electrochemical cell. Water electrolysis is less efficient in practice, requiring external potentials much above the thermodynamic minimum of 1.23 V vs. RHE. The overpotential is required to accelerate electron transfer activities at high rates and overcome kinetic barriers provided by high activation energies to produce chemical intermediates on the electrode surface. Electrocatalysts with low overpotentials for the water splitting reactions are known as efficient electrocatalysts (Morales-Guio et al., 2014). The current density at a given voltage is frequently used to evaluate the performance of electrocatalysts (Xiong et al., 2018). Furthermore, hydrogen is regarded as the most environmentally friendly renewable energy source and the main replacement for fossil fuels in the event of a future energy shortage. A significant requirement for the realization of a future hydrogen economy is sustainable hydrogen generation. The electrochemical HER, which is an essential stage in the process of producing H_2_ from water electrolysis, has been the focus of much research in recent decades. Literature significantly reflects numerous research advancement followed by peer reviewed article has been published in this research area. Also, by considering the future prospective efficient electrocatalyst innovation in technology and large-scale production the studies need to be summarized for upcoming researchers.

Expanding on the previous study, this comprehensive overview explains the latest state-of-the-art advances in catalyst technology by combining the fundamental ideas of Hydrogen Evolution Reaction (HER) and Oxygen Evolution Reaction (OER). This review discusses about the developments in low-cost and high-performance catalysts, including metal-based and metal-free HER and OER electrocatalysts, as well as noble and non-noble metals. The review methodically investigates the complex relationships between composition, morphology, structure, and catalytic active sites as well as synthetic techniques. Also, present a comprehensive analysis of catalyst efficiency-boosting measures, including raising the intrinsic activity of active sites and increasing the total number of active sites.

### 2.1 Electrocatalytic hydrogen evolution reactions (HER)

HER is a multi-step reaction in which two-electron transfer takes place on cathode via. Two different mechanisms with three possible reactions, i.e., the first reaction is Volmer reaction in this reaction electrochemical hydrogen adsorption can takes place, the second reaction is Heyrovsky reaction in which electrochemical desorption of hydrogen occurs, and the third one is Tafel reaction in this reaction chemical desorption of hydrogen molecule, i.e., H_2_ is eliminated. The electrochemical HER process involves three probable major phases for the reduction of hydrogen in acidic condition or water molecules in alkaline condition to hydrogen (H_2_) molecules on the surface of an electrode with a minimal external applied voltage (Morales-Guio et al., 2014; Zhu et al., 2020). In the Volmer reaction (Eqs 6, 7) is the initial step, in which a proton combines with an electron to produce an adsorbed hydrogen atom (H*) on the electrode surface (M). In acidic and alkaline electrolytes, the proton sources are the hydronium cation (H_3_O^+^) and the water molecule respectively. Following that, H_2_ may be formed by the Heyrovsky reaction (Eqs 8, 9) or the Tafel reaction (Eq. 10), or both. In the Heyrovsky reaction, another hydrogen diffuses to the H* and then interacts with a second electron to generate H_2_ molecule. Whereas, in the Tafel step, two H* in the vicinity combine on the surface of the electrode to evolve H_2_. The overall HER can be written as follows:

1) Electrochemical Hydrogen Adsorption (Volmer Reaction)

$$H_{3}O^{+}{+M+e}^{‐}\;⇋\;M\;‐{\;H*+H}_{2}O\;\left(\text{acidic medium}\right)$$ (6)

$$H_{2}{O+M+e}^{‐}\;⇋\;M\;‐{\;H*+\text{OH}}^{‐}\;\left(\text{alkaline medium}\right)$$ (7)

2) Electrochemical Desorption (Heyrovsky Reaction)

$$H^{+}{+e}^{‐}+M\;–\;H*\;⇋{\;H}_{2}+M\;\left(\text{acidic medium}\right)$$ (8)

$$H_{2}{O+e}^{‐}+M\;–\;H*\;⇋{\;H}_{2}{+\text{OH}}^{‐}+M\;\left(\text{alkaline medium}\right)$$ (9)

3) Chemical Desorption (Tafel Reaction)

$$2M\;–\;H*\;⇋{\;H}_{2}+2M\;\left(\text{both acidic and alkaline medium}\right)$$ (10)

The HER in an alkaline environment is sluggish the reaction than in an acidic environment due to water dissociation before the generation of H*. H* is constantly active in the HER, regardless of the paths via which it progresses. As a result, by thermodynamic point of view, the free energy of hydrogen adsorption (∆GH*) is commonly used as a symbol for hydrogen-evolving materials. ∆GH* of an ideal electro-catalyst for the HER, such as Pt, should be near to zero since weak adsorption leads to poor contacts between hydrogen and electrode surfaces, but ∆GH* is too big to break hydrogen-catalyst surface connections, preventing H_2_ desorption. The volcano relationship of a wide variety of catalysts estimated from density functional theory (DFT) vs. the logarithm of their corresponding exchange current densities (log *j*_0_), which shows the HER activities of various metals, is empirically displayed using ∆GH*. The so-called volcano diagram provides a simple method to view and compare the activities of various metals, which can aid in material design optimization. The Tafel slope, which is an intrinsic feature of an electrocatalyst and can be acquired from a Tafel plot produced from the polarization curve, may be used to assume the HER process (Anantharaj et al., 2016; Li et al., 2016). The Volmer, Heyrovsky, and Tafel reactions have Tafel slopes of 118 mV/December, 39 mV/dec, and 29 mV/dec, respectively, under typical conditions. The Tafel slope of a Pt electrode on the Pt (110) plane for the HER is found to be 30 mV/dec, indicating that the rate-determining step (RDS) of Tafel reaction. However, the rate-determining step for a Pt electrode with a Pt (100) plane might be the Heyrovsky reaction Nanocomposites are created utilizing nanoparticles (NPS), which offer benefits in terms of multifunctionality, economics, and environmental issues. In this study, both inorganic and catalytic uses of nanomaterials as in heterostructured electrocatalysis performed water-splitting processes, structural flaws of such effective ideal catalysts have recently been exploited as heteroatomic doping and multi-functional catalysts for HER and OER. Further, the electrocatalysts employed in the Oxygen Reduction Reaction (ORR) and OER have been proven to work well in metal/air batteries and also been reported that to utilize the urea oxidation reaction (UOR), which includes a six-electron transfer reaction as an electrochemical reaction, high-efficiency catalysts are required. This review should be applicable to the long-lasting and diversified area of electrochemistry (Jang et al., 2020). As an electrocatalyst’s Tafel slope is 30 mV/dec could not be created only by the Tafel reaction. In general, the coverage state of an adsorbed hydrogen atom on the surface of the electrode materials should be connected to either the Tafel RDS or the Heyrovsky RDS. Furthermore, the Tafel slope is affected by applied voltage and temperature. As a result, it is critical to grasp the fundamental method of calculating and evaluating the Tafel slope. Furthermore, due to the inflated reading of the Tafel transcript, Tafel’s reaction will be misconstrued (Shinagawa et al., 2015).

Based on their physical and chemical characteristics, the components used to produce HER electrocatalysts are categorized into three groups: (i) Noble metal Platinum (Pt) is used as the sophistication of HER electrocatalysts; (ii) Transition metals, such as iron (Fe), cobalt (Co.), nickel (Ni), copper (Cu), molybdenum (Mo), and tungsten (W), are used to form noble metal-free HER electrocatalysts; (iii) Non-metals, such as boron (B), carbon (C), nitrogen (Se). Until now, the above twelve non-precious elements have been used to create efficient noble metal-free HER electrocatalysts. In weight percent, the earth’s crust abundance of the metals used to make HER electrocatalysts. The following conclusions can be derived by comparing their quantities in the shell: (i) Pt has a lower abundance than any other non-precious metal, at about 3.7106% and has most costly on the planet in this category. (ii) The abundance of non-noble metals rises in the sequence Mo < Co. < Cu < Ni << Fe. It is crucial to take in mind the variations in abundance and possible cost while constructing non-noble metal HER catalysts.

### 2.2 Electrocatalytic oxygen evolution reactions (OER)

The OER is a four-electron-transfer reaction that produces molecular oxygen at anode. Interestingly, four hydroxyl ions are oxidized into two water molecules and one oxygen molecule in alkaline and neutral electrolytes whereas in acidic electrolytes four water molecules are oxidized into four protons and one oxygen molecule. Due to the slow reaction kinetics, and reaction pathways of the OER having more complicated than the HER. The three adsorbed intermediates, i.e., OH*, O*, and OOH* on the catalytic surface are commonly involved in the assumed OER processes. All supposed pathways for the formation of OH* in alkaline electrolyte include an important elementary stage of hydroxide coordination to the active sites and a subsequent elementary step of the formation of additional intermediates. The suggested OER processes all have the same intermediates, such as OH* and O*, regardless of the reaction circumstances, with the generation of oxygen being the key variation. The atomistic knowledge of the OER process serves as the foundation for a descriptor-based approach for the overall activity. The commonly recognized processes for the OER in acidic and alkaline environments can be presented individually in four steps, as detailed in Eqs 11–14 or Eqs 15–18, based on the theoretically presented models (Man et al., 2011; Hong et al., 2015; Suen et al., 2017; Wang et al., 2020). However, the mechanistic pathway of OER in alkaline as well as acidic electrolyte has been below:

OER in acidic electrolyte:

$$H_{2}{O+*\;\to \;\text{HO}*+H}^{+}{+e}^{‐}$$ (11)

$${\text{HO}*\;\to \;O*+H}^{+}{+e}^{‐}$$ (12)

$${O*+H}_{2}{O\;\to \;\text{HOO}*+H}^{+}{+e}^{‐}$$ (13)

$${\text{HOO}*\;\to \;*+O}_{2}{+H}^{+}{+e}^{‐}$$ (14)

OER in alkaline electrolyte:

$${4\text{OH}}^{‐}{\;\to \;\text{OH}*+3\text{OH}}^{‐}{+e}^{‐}$$ (15)

$${\text{OH}*+3\text{OH}}^{‐}{\;\to \;O*+2\text{OH}}^{‐}{+H}_{2}{O+e}^{‐}$$ (16)

$${O*+2\text{OH}}^{‐}{+H}_{2}{O\;\to \;\text{OOH}*+\text{OH}}^{‐}{+H}_{2}{O+e}^{‐}$$ (17)

$${\text{OOH}*+\text{OH}}^{‐}{+H}_{2}{O\;\to \;O}_{2}{+2H}_{2}{O+e}^{‐}$$ (18)

The observed electrode potential vs. the standard hydrogen electrode (SHE) is 0 under standard circumstances, and the theoretical overpotential for OER has the following connection with ∆G_OER_ (Joo et al., 2019):

$$\eta_{\text{OER}}=\left(\frac{{∆G}_{\text{OER}}}{e^{‐}}\right)–\;1.23V$$ (19)

For η_OER_ = 0, ∆G_OER_ equals 1.23 eV, meaning that the Gibbs free energy of each step for an ideal OER catalyst is 1.23 eV. Thus, the volcano plots for OER demonstrated that the over-potentials of a wide variety of metal oxides vs. (∆G^0^O* - ∆G^0^HO*) have been plotted, providing some hints for the design and optimization of highly efficient OER catalysts (She et al., 2017). With extremely strong and very weak oxygen bonding forces creating delayed reactions, ∆GO* - ∆GHO* may also be utilized to measure catalytic activity. Meanwhile, the Tafel slope is connected to the overpotential, which has helped many researchers to understand the OER chemical mechanism (Bockris, 1956; Matsumoto and Sato, 1986). To summarize, ∆G_OER_ can offer thermodynamic information between intermediates and catalysts, whereas the Tafel slope can provide kinetic information about catalysts in OER reactions under experimental conditions.

### 2.3 Role of electrolytes in water-splitting reactions

The role of size plays an important role in water-splitting processes, particularly in electrolysis, which can affect the process’s selectivity and efficiency. Water splitting in electrolysis happens at the electrodes, where the application of an electric current splits water molecules (H2O) into hydrogen (H_2_) and oxygen (O_2_) gas. Electrolytes are important in water-splitting reactions like electrolysis because they allow ions to travel between the electrodes and the electrolyte more easily. They improve the overall reactions efficiency and aid in keeping the system’s charge neutrality (Bard et al., 2022). Electrolytes play the following important functions in water-splitting reactions:

i. Ionic Conduction: Ions in electrolytes are free to circulate throughout the solution. The water-splitting reaction is made possible by the charge transfer between the electrodes made possible by this ionic conduction. Ion flow would be severely restricted in the absence of electrolytes, resulting in poor conductivity and ineffective reactions.
ii. Redox processes: Electrolytes are frequently involved in redox (reduction-oxidation) processes, which help turn water into gaseous hydrogen and oxygen. For instance, in acidic electrolytes, hydroxide ions (OH^−^) produced at the anode may undergo oxidation, whereas protons (H^+^) from the electrolyte solution may take part in the reduction process at the cathode.
iii. Electrode Stability: During the water-splitting reaction, electrolytes can have an impact on how stable the electrodes are. By creating a barrier or regulating the solution’s pH, they can aid in preventing the electrodes from corroding. For electrolysis cells to function well over the long term, this is very crucial.
iv. Product Selectivity: The electrodes’ ability to selectively bind to the intended products (oxygen and hydrogen) can also be influenced by the electrolyte selection. The production of hydrogen or oxygen gas can be facilitated by certain electrolytes, contingent on their pH and content.
v. Electrolyte Composition: The kinetics and effectiveness of the water-splitting reaction can be affected by the electrolyte’s composition. To increase conductivity and reaction speeds, for instance, certain salts or acids might be added to the electrolyte solution.
vi. Size of Cation: The electrolyte solution’s cation, or positively charged ion, impacts how quickly water splits. Because of their size, larger cations could find it more difficult to access the electrode surface, which would slow down reaction rates. On the other hand, smaller cations have an easier time approaching the electrode, which speeds up the reaction rate.
vii. Anion Size: Similarly, the water-splitting process may also be impacted by the anion’s (a negatively charged ion) size. The process may be slowed down by larger anions obstructing the flow of water molecules towards the electrode surface. Conversely, smaller anions may make it easier for water molecules to move around, which might speed up the process.
viii. Electrode Selectivity: The electrodes’ ability to selectively attract the intended products (H_2_ and O_2_) can also be influenced by the size of the ions. For instance, smaller cations may adsorb more firmly on the electrode surface during the oxygen evolution reaction (OER), which would encourage the production of oxygen gas. Larger cations, on the other hand, could preferentially adsorb and produce hydrogen gas as a result.
ix. Electrolyte Conductivity: The conductivity of the electrolyte solution can be affected by ion size. Bigger ions may make it more difficult for charge carriers to travel, which would lower the electrolyte’s total conductivity. Since quicker and more efficient reactions are often associated with better conductivity, this might have an impact on the electrolysis process’ efficiency.

Overall, since they facilitate the transfer of charge, stabilize the electrodes, and affect the reaction’s selectivity, electrolytes are crucial parts of water-splitting processes. Researchers can enhance electrolysis procedures for effective and sustainable hydrogen generation by comprehending the significance of electrolytes. In addition, the efficiency, selectivity, and kinetics of water-splitting processes in electrolysis may all be significantly influenced by the size of the ions present in the electrolyte solution. Hydrogen generation and energy storage are two uses for electrolysis processes that may be optimized with the assistance of an understanding of and control of ion size effects.

## 3 Electroanalytical insights: methods for evaluating catalytic performance

Prior to exploring the advanced development of catalysts, it is critical to define important assessment criteria. These characteristics are essential for evaluating how well the catalyst splits water. The extensive set of parameters comprises the following: electrochemical active surface area (ECSA) (Jung et al., 2016), chronoamperometry (CA) (Aoki et al., 1985), turnover frequency (TOF) (Wang et al., 2018), solution resistance, charge transfer resistance (Bredar et al., 2020), Tafel slope (Shinagawa et al., 2015), onset potential, overpotential (Appel and Helm, 2014), current density, and faradaic efficiency (FE) (Jung et al., 2016). The two-electrode method and the traditional three-electrode method are both used to assess the performance of water splitting. These experimental configurations make it possible to evaluate the water-splitting process’ efficacy and efficiency in great detail. A working electrode, a reference electrode, and a counter electrode are used in a typical three-electrode setup. The reference electrode keeps an eye on the electrochemical potential, the counter electrode completes the electrical circuit, and the working electrode is in charge of catalyzing the water-splitting reaction. This technology enables a thorough investigation of the performances of each electrode and offers exact control over the reaction circumstances.

However, a typical electrochemical system consists of two electrodes, i.e., one is the working electrode and other one is a counter electrode, which also acts as the reference electrode. This setup is easier to understand and is frequently used in situations when a more basic structure is appropriate. It may not be as precise as the three-electrode technique, but in specific experimental situations, it has advantages. Researchers may obtain a thorough grasp of the water-splitting performance under various situations by employing both the three-electrode and two-electrode systems. This allows for a more thorough evaluation of catalyst efficiency and overall system efficacy. A working electrode can be made of a variety of materials, including glassy carbon electrodes, Ti foil, porous metal foams (Fe, Ni foam), carbon cloth, or carbon paper in a traditional three-electrode system that consists of a working electrode, a reference electrode, and a counter electrode. The reversible hydrogen electrode (RHE), Ag/AgCl, saturated calomel electrode (SCE), and Hg−HgO electrode are among the alternatives for the reference electrode, which is essential for stability during catalysis. The working and reference electrodes are linked via a salt bridge utilizing a lugging capillary tip to ensure precision.

Reference electrode selection depends on the pH and takes into account elements such as electrolyte alkalinity. Saturated calomel or Ag/AgCl electrodes, for example, could not be appropriate for alkaline electrolytes. Nonetheless, reliable operation for extended periods of time without accuracy loss may be ensured by connecting the reference electrode to the working electrode via a double salt-bridge, preventing direct contact with the alkaline electrolyte (Feng et al., 2014). Usually composed of inert materials such as Pt, Au, graphite, or glassy carbon, the counter electrode-also referred to as the auxiliary electrode-ensures that it is unaffected by the electrochemical process. It is important to remember that cathodic deposition of Pt or Au counter electrodes may affect the results of the HER test. The counter electrode functions as both the reference electrode and the reference potential in a two-electrode system in which no separate reference electrode is employed. The catalytic system efficiency in actual devices is best represented by this system. It is crucial to keep in mind that the parameters listed in the literature are directly related to test setups and procedures, especially when it comes to setting up the working electrode.

### 3.1 Current density

In electrochemical measurements, assessing current density involves different strategies based on the type of working support electrode. Two common approaches include using conventional electrodes, such as glassy carbon or Ti foil, and employing porous conductive supports like metal foams (Fe, Ni foam), carbon cloth, or carbon paper. The choice significantly impacts measurement accuracy and reliability. For conventional electrodes, the measurable and reliable electrode surface remains inert during catalytic reactions. Performance, expressed as current normalized to the geometric surface area, remains consistent. In contrast, porous electrodes lack a well-estimated surface area due to their non-planar structure. This can lead to underestimated surfaces, resulting in reported performances significantly higher than those on inert electrodes.

When catalysts are self-supported on porous electrodes, variations in immersed dimensions occur during fabrication. Despite efforts to seal undesired surfaces, changes persist. The interaction between support and catalyst enhances catalytic performance. Powder catalysts on inert glassy carbon electrodes are preferable for fundamental studies, where current normalized to geometric surface area allows fair comparisons. The mass current density becomes relevant when uncertainties in surface area exist, especially for self-supported catalysts. For a standardized comparison, it is crucial to use consistent catalyst loading during ink preparation. While lower amounts may exhibit higher mass current density, higher amounts may maintain similar geometrical current with reduced mass current density. In evaluating catalyst efficiency, specific activity, calculated by normalizing raw current to the electrochemical surface area (ECSA), provides fairness for different catalyst systems. The challenge lies in ECSA determination, where techniques like electrochemical double-layer capacitance and BET surface area are employed. Standardized ECSA calculation methods would enhance convincing and fair catalyst comparisons.

### 3.2 Overpotential (*η*)

In the context of the water-splitting process, each half-reaction is propelled forward by its thermodynamic potential. Specifically, the thermodynamic potentials for the HER and OER are 0 V and 1.23 V vs. RHE, respectively, with respect to a standard hydrogen electrode. There is a significant discrepancy between the expected and observed values for the standard free energy change associated with the basic stages in these reactions, even though the values are theoretically predicted. Overpotential (η) is the term used to describe the considerable departure of potential seen in experimental results from the thermodynamically expected value. Overpotential arises due to various factors such as kinetic barriers, transport limitations, and other impediments in the reaction pathway.

Presently, the overpotential necessary to achieve a current density of 10 mA/cm^2^ serves as a key metric for assessing the catalytic efficacy of a novel catalyst. This pivotal performance indicator was established based on the anticipated current density in a solar-to-fuels integrated device operating under one Sun illumination with a 10% solar-to-fuels efficiency (Kibsgaard et al., 2014). The geometric area of the electrode determines the current density, which makes catalysts applied to an inert support electrode with a precisely determined surface area more appropriate for these requirements. Ideally, a catalysts capability is higher when the overpotential required for achieving this current density is lower.

Because the surface area of catalysts supported or grown on the second type of electrode is usually overestimated, a greater current density is usually seen. As a result, overpotentials at high current densities (such as 50 and 100 mA/cm^2^) have been suggested as substitute metrics for activity assessment (Chen et al., 2013). As was previously said, a legitimate and accurately measured surface area is necessary for a fair comparison. The overpotential needed to reach this benchmark current density in the back-scan direction can also be used as a comparison for materials displaying a prominent redox peak in the Oxygen Evolution Reaction (OER), where the oxidation current surpasses 10 mA/cm^2^ in the positive scan direction within the potential window of gas release. It is important to remember that this current density only applies to the splitting of water. It is crucial to highlight that this current density is specifically relevant to the water-splitting reaction, signifying a 100% current efficiency for the electrochemical process.

### 3.3 Tafel slope

The Tafel equation serves as a fundamental expression in electrochemical kinetics, establishing a connection between the rate of an electrochemical reaction and the overpotential. A simplified form of the equation is $\eta =a+b\;\log\left(j\right)$ , where *η* represents the overpotential, and *b* is the Tafel slope. The Tafel slope (*b*) is pivotal for understanding the reaction mechanism, while the exchange current density provides insights into catalytic activity (Wang et al., 2016). The exchange current density, denoting the current in the absence of net electrolysis and at zero overpotential, plays a crucial role in assessing catalyst kinetics. It is obtained from the Tafel plot by extrapolating the current to V = 0, signifying the rate of oxidation or reduction at equilibrium. A higher exchange current density indicates a faster reaction, emphasizing swift electron transfer processes at the electrode/electrolyte interface.

Typically, the Hydrogen Evolution Reaction (HER) entails three elementary reactions, each associated with a characteristic Tafel slope. The initial step, known as the Volmer reaction, is represented as:

$$H_{\text{ads}}^{+}{+e}^{‐}\;\to \;H_{\text{ads}}$$ (20)

This reaction exhibits a slope value of 118 mV/dec. In this step, an electron is transferred to a hydrogen proton attached to the catalyst, leading to the formation of adsorbed hydrogen (H_ads_) (Wang et al., 2015). In the subsequent step, termed the Heyrovsky reaction, the adsorbed H_ads_ tends to combine with a new electron and another hydrogen to produce H_2_ molecules at a low H_ads_ coverage:

$$H_{\text{ads}}+H_{\left(\text{aq}\right)}^{+}{+e}^{‐}\;\to \;H_{2\;\left(g\right)}$$ (21)

This process is called as the Heyrovsky reaction, and the Tafel slope value is 39 mV/dec. If the H_ads_ coverage is high, the dominant process involves recombination between adjacent H_ads_ atoms, leading to the generation of H_2_.

$$H_{\text{ads}}+H_{ads}\;\to \;H_{2\;\left(g\right)}$$ (22)

The Tafel slope value of a typical Tafel process, which is suggested, is 29.5 mV/dec. Desorption of H_2_ molecules from the catalysts is the final stage. The proton availability is dependent on the electrolyte’s pH: in acidic media, the catalyst may extract hydrogen protons from the electrolyte, but in neutral and alkaline media, the proton generation mostly comes from H−O−H bond breaking of H_2_O (Zhang et al., 2019).

The following reaction stages have been postulated for the OER process, where M stands for metal, aq for dissociating in aqueous solution, and ads for an adsorption state (Feng et al., 2020):

$$M+OH_{\left(\text{aq}\right)}^{‐}\;↔\;{M‐\text{OH}}_{\left(g\right)}{+e}^{‐}$$ (23)

$$M‐\text{OH}+OH_{\left(\text{aq}\right)}^{‐}{\;↔\;M‐O+H}_{2}{O+e}^{‐}$$ (24)

$$M‐O+H_{\left(\text{aq}\right)}^{‐}\;↔\;{M‐\text{OOH}}_{\;\left(\text{ads}\right)}{+e}^{‐}$$ (25)

$$M‐\text{OOH}+{\text{OH}}_{\left(\text{aq}\right)}^{‐}\;\to \;{\text{MOO}}_{\left(\text{ads}\right)}^{‐}{+H}_{2}O$$ (26)

$${M‐\text{OO}}^{‐}\;↔\;M+{\;O}_{2}{+e}^{‐}$$ (27)

If the initial electron transfer reaction serves as the rate-determining step (RDS), the corresponding Tafel slope (denoted as b) is 118 mV/dec, resembling that of a single electron transfer reaction. In this context, the surface intermediate species that emerges right before the Rate-Determining Step (RDS) takes precedence. When the RDS encompasses the chemical reaction subsequent to a one-electron transfer, the Tafel slope experiences a reduction to 59 mV/dec. If the RDS pertains to the third electron transfer reaction, the Tafel slope value further decreases to 29.5 mV/dec (Shinagawa et al., 2015).

Despite the frequent use of the Tafel slope as the primary activity parameter in determining catalytic activity, there are inherent challenges in its acquisition. Typically, the Tafel plot is constructed by converting the linear sweep voltammetry (LSV) curve, which may lead to misconceptions about catalytic activity. It is imperative to note that LSV must be conducted under steady-state conditions to minimize experimental error. Even at a scan rate of 1 mV/s, the current obtained in LSV may not represent a true steady-state current. While, for the sake of experimental efficiency, a scan rate of 5 mV/s or below is commonly used, resulting in a Tafel slope that remains very close. To accurately represent variations in current that are governed by kinetics, linear fitting has to be limited to the linear portion of the Tafel range. If the current density is excessively high, surpassing 20 mA/cm^2^, polarization may be governed by concentration polarization, and the obtained slope may fail to reflect kinetic information. An alternative and more accurate method for determining the Tafel slope is through electrochemical impedance spectroscopy (EIS) (Liu et al., 2020a). This method involves the regular acquisition of Nyquist plots of the catalysts at different overpotentials, from which the Tafel slope may be calculated by fitting the plot of log(*R*_*ct*_) vs. *η*, where *R*_*ct*_ is the charge transfer resistance. This technique determines the catalyst’s precise Tafel slope based only on its capacity for charge transfer.

### 3.4 Uncompensated solution resistance and charge transfer resistance

In conventional electrochemical studies, the presence of uncompensated solution resistance (*R*_*u*_) in electrolyte cells is predominantly governed by the cell geometry and the composition of the electrolyte. This resistance is influenced by various factors such as the shape and size of the cell, the positioning of the reference electrode, the configuration of the working electrode, and the dimensions of the counter electrode (Myland and Oldham, 2000). The manifestation of *R*_*u*_ can be troublesome, causing significant errors in the measured potential of the cell. To attain an accurate polarization curve, an *iR* correction is imperative. This correction can be automatically executed using built-in or post-correction approaches once the *R*_*u*_ value is known. A practical method for *iR* correction is the current interrupt technique, which is efficiently employed in many commercial electrochemical workstations. These workstations utilize a circuit to momentarily interrupt the current flow through the cell, typically for a few milliseconds. The cell voltage is then measured both before and after the current interruption, and the voltage difference that results is known as the uncompensated voltage (*V*_*u*_). This voltage difference is then used to dynamically adjust the observed potential. The current interrupt method possesses an advantage as *R*_*u*_ need not be determined beforehand. Its dynamic correction capability mitigates errors arising from *R*_*u*_ fluctuations during experiments.

In contrast, positive-feedback of *iR* compensation is a post-calibration approach requiring the prior for the determination of *R*_*u*_, typically obtained from electrochemical impedance spectroscopy (EIS). Electrochemical workstations often provide this calibration approach, where the user inputs Ru and sets a compensation percentage (usually 80–90%). A portion of the current signal is sent back as an extra voltage input when positive feedback is engaged. The Ru value can also be used to manually complete this calibration. Positive-feedback *iR* correction facilitates voltage feedback, ensuring reliable correction. It is noteworthy that similar electrode configurations exhibit similar *R*_*u*_ values. For instance, our group reported *R*_*u*_ values around 7–8 Ω for a catalyst-coated glassy carbon electrode used as the working electrode (Liu et al., 2020b).

A comparable resistance to electrolyte resistance is the charge transfer resistance (*R*_*ct*_), arising from a single kinetically controlled electrochemical reaction. It represents the resistance encountered during the transfer of electrons from one phase (e.g., electrode) to another (e.g., liquid). EIS, particularly the Nyquist plot, is instrumental in determining *R*_*ct*_ by fitting it with an appropriate equivalent circuit. The value of *R*_*ct*_ is proportionate to the diameter of the semicircle or arc in the Nyquist plot, offering a direct insight into the charge transfer ability. The Nyquist plot, illustrates ideal plots for catalysts driving reactions at different potentials or different catalyst comparisons under identical conditions. Based on the theory of the EIS (Chang and Park, 2010), at high frequency, the relationship between *Z*_Re_ and *Z*_Im_ becomes ${\left(Z_{\text{Re}}‐R_{\Omega }‐\;\frac{R_{\text{ct}}}{2}\right)}^{2}+Z_{\text{Im}}^{2}={\left(\frac{R_{\text{ct}}}{2}\right)}^{2}$ and thus a characteristic arc shape is observed in the Nyquist plot. The radius of this arc is $\frac{R_{\text{ct}}}{2}$ . In the high-frequency band, the impedance spectrum takes on a distinct arc form, with *Z*_*Re*_ representing the uncompensated solution resistance. In the low-frequency range, *Z*_*Re*_ is the product of charge transfer resistance $\left(R_{u}{+R}_{ct}\right)$ and uncompensated solution resistance. The impedance spectrum can be acquired under open-circuit conditions or while running a reaction at a certain current or potential to drive the reaction (Bredar et al., 2020). *R*_ct_ is reported under both these conditions, with the open-circuit condition reflecting charge transfer capability, while the running condition is preferred for probing kinetics.

### 3.5 Electrochemical active surface area (ECSA) and roughness factor

The importance of achieving a high surface area in catalysts, which exposes more active sites, cannot be overstated for enhancing catalytic activity. Although the Brunauer−Emmett−Teller (BET) N_2_-adsorption method is a widely used technique for assessing the specific surface area of a porous catalyst, it is essential to approach the results with caution. The surface area determined by BET may not precisely represent the electrochemical surface area of an electrocatalyst. In the case of electrocatalysts, it is the electrochemical surface area that interacts with the electrolyte to facilitate mass and charge transfer, making it a pivotal factor for efficient electrochemical reactions. Consequently, electrochemical techniques are better suited to evaluate the surface area of catalysts, and the Electrochemical Double-Layer Capacitance (C_dl_) is extensively used for this purpose. The C_dl_ can be calculated from cyclic voltammetry (CV) curves obtained in a specific potential window without Faradaic processes (Ma et al., 2017).

Generally, five to ten successive CV curves are obtained at varying sweep rates (5–200 mV/s), and the resulting curves—ideally near a rectangular shape—are chosen. The difference in current density under that potential is computed (Δ*j* = *j*_1_ − *j*_2_, or the sum of the absolute value of the current), once the intermediate value of the applied potential range has been determined. A plot with different sweep speeds as the *X*-axis and Δ*j* as the *Y*-axis is subjected to linear fitting to obtain the slope, which is twice the value of C_dl_. A larger slope indicates a higher surface area with more exposed surface reactive sites (Li et al., 2018). To calculate ECSA, a specific capacitance, typically C_S_ = 40 μF/cm^2^ for a smooth flat metal surface, is used as a standard reference. Subsequently, the ECSA is then obtained through the formula ECSA = C_dl_/C_S_, where C_dl_ represents the double-layer capacitance, and CS is the specific capacitance. The roughness factor (F), calculated as the ratio of electrochemically active surface area (ECSA) to the geometric surface area (S_geo_) of the working electrode (F = ECSA/S_geo_), is determined. This method ensures fair and acceptable comparison of specific activities among different catalysts.

### 3.6 Evaluation of stability

In practical applications, electrocatalyst stability is crucial and involves dynamic and steady-state stability issues. To evaluate dynamic stability, the catalyst is put through accelerated deterioration tests, accomplished by cycling cyclic voltammetry (CV) at a high scan rate. The potential window is limited within the kinetic potential range, permitting a current density range of 0–20 mA/cm^2^ to assess kinetic stability. Excessive gas product-induced electrode separation and mass transfer are avoided by exercising caution when surpassing greater current densities. Polarization data is collected after the accelerated deterioration test and is compared to the plot from the pre-stability test. Stability is measured by characteristics like the shift of the onset overpotential (η_0_) and the overpotential at a particular current density (e.g., 10 mA/cm^2^, η_10_); a more stable catalyst will respond favourable to these parameters. Conversely, steady-state stability is usually studied using chronoamperometric or chronopotentiometric measurements carried out over a prolonged period with an applied potential or current density that remains constant. The current density and applied voltage selected are meant to maintain a current density between 10–20 mA/cm^2^. It is customary to use a 10-h assessment time as a benchmark for the perfect catalyst. Because of the production and elimination of gas products from the electrode, there may be minute variations in current or potential during the test (McCrory et al., 2015).

### 3.7 Turnover frequency (TOF) and catalytic activity evaluation

The concept of Turnover Frequency (TOF) serves as a valuable metric for characterizing catalyst performance, defining the number of reactants converted into the desired product per unit of catalytic active site in a unit of time. However, the precise number of active sites in an electrocatalyst remains unclear, leading to an underestimation of TOF. To calculate TOF (s^−1^) for the Hydrogen Evolution Reaction (HER), the formula $\text{TOF}=I/\left(2\text{Fn}\right)$ (Yang et al., 2019) is employed, where I represents the current (A) during linear sweep measurement, F is Faraday’s constant (96485.3 C/mol), and n denotes the number of active sites (mol). The two electrons needed to make one hydrogen molecule are taken into consideration by factor 1/2. Similarly, for the Oxygen Evolution Reaction (OER), the TOF (s^−1^) is calculated using $\text{TOF}=I/\left(4\text{Fn}\right)$ , taking into account that one oxygen molecule requires four electrons.

### 3.8 Faradaic efficiency

Assessing the Faradaic Efficiency is crucial for evaluating the water-splitting reaction. Various techniques can be employed, with a direct approach involving a comparison between theoretically predicted and experimentally obtained products. Faradic efficiency is evaluated by applying a constant potential or current to the working electrode and the gas produced is compared to the theoretical quantity to evaluate the efficiency (Anantharaj et al., 2018). A simple and cost-effective method involves collecting gas products over water during catalysis, utilizing a gas collection container filled with water inverted in a water reservoir above the electrode (Chen et al., 2020). The evolved gas displaces water from the inverted container, and the gas volume is measured using a graduated cylinder. This approach, while economical, may introduce experimental errors, especially for lower gas amounts in laboratory-scale setups.

For more accurate measurements, real-time monitoring of gas amounts can be achieved using instruments like gas chromatograph (GC) or gas sensors. This method requires a sealed electrochemical cell, introducing complexity compared to the water collection method. GC, commonly used for measuring gas amounts and purity, involves continuous injection of the generated gas into the instrument (Dotan et al., 2019). A sealed electrochemical cell can be fitted with a gas sensor or pressure sensor to record the quantity of gas created if a GC is not accessible. For instance, linear tracking of gas output over time is possible using a gastight electrochemical cell and pressure sensor. By utilizing the ideal-gas formula PV = nRT, one may ascertain the volume of the experimental gas and subsequently compute the faradic efficiency by contrasting it with the volume of the theoretical gas. The formula is used to determine the faradic yield, which is the ratio of the measured gas volume to the predicted gas volume (McCrory et al., 2015):

$$\text{Faradaic efficiency}=\frac{V_{\text{experimental}}}{V_{\text{theoretical}}}=\frac{V_{\text{experimental}}}{\frac{1}{2\left(4\right)}\;\frac{Q}{F}\text{ Vm}}$$ (28)

where F is Faraday’s constant (96485.3 C/mol), Q is the charge that passes through the electrode, and Vm is the molar volume of gas (24.5 L mol^−1^, 298 K, 101 KPa). The numbers 2 and 4 denote the number of electrons per mole of H_2_ and O_2_, respectively.

## 4 Overall noble metal-free systems for HER and OER and their merits and demerits

Heteroatom-doped carbon compounds have additional benefits over metal doped carbon nanostructured water electrolysis due to their inexpensive cost, high electrical conductivity, molecular architecture, abundance, high catalytic efficiency, and high acid/alkaline tolerance. Overdoped carbon materials have recently attracted a lot of interest, and they have been recognized as a bottleneck for HER catalytic efficiency. When carbon atoms are co-doped with numerous heteroatoms (such as N, P, S, or O), different electro-negativities than carbon are produced, potentially enhancing electrocatalytic water splitting activities through a synergistic effect when compared to single heteroatom-doped analogues (Xiong et al., 2018). More crucially, by varying doping types, locations, and amounts, these catalysts demonstrate tailorable catalytic capabilities for various electrocatalytic processes followed by OER application. The electrocatalytic activity of metal-free heteroatom-doped carbon-based nanomaterials is primarily governed by three factors such as: (1) the intrinsic nature of active sites, which is dictated by chemical composition and interactions between different components; (2) the specific surface area and existence of hierarchically porous structure, which can considerably impact the accessibility of active sites and reaction transport qualities to significant species; and (3) the electron transfer energy, which is defined by the electrical conductivity of the catalyst and its binder-free structure. Finally, carbon-based materials have fulfilled with characteristic properties as an electrocatalysts to showing water splitting that exhibit the three features described above and will work well in water electrolysis operations (Lai et al., 2016).

Literature significantly reflects, Lai et al. were the first to report on metal free heteroatom (N, O, and P) tri-doped porous graphitic carbon on oxidized carbon fabric for water splitting electrocatalyst with preparation employing aniline, phatic acid, and oxidized carbon cloth as a precursor. With a cell potential of 1.66 V vs. SCE and a current of 10 mA/cm^2^, it functions well in alkaline electrolytes. The abundance of N, O, and P in metal-free nanocarbon provides abundance of active sites for HER and OER (Lai et al., 2016). Dai’s group proposed a simple pyrolysis technique using polyaniline (PANI) coated graphene oxide (GO-PANI) to construct metal-free N, P, and F tri-doped graphene electrocatalysts for ORR, OER, HER, and Zn-Air batteries in the presence of ammonium hexafluorophosphate (AHF). As a multifunctional catalyst for self-powered electrochemical water splitting, graphene is tridoped with nitrogen, phosphorus, and fluorine (Zhang and Dai, 2016). Non-metal NC electrocatalysts showed the lowest overpotential for OER in alkaline solution, according to Zhao et al., and were the first non-metal electrocatalysts for the water-splitting process (Zhao et al., 2013). Nitrogen-doped carbon compounds are effective oxygen evolution electrocatalysts. In an alkaline solution, the material generated a current density of 10 mA/cm^2^ with an overpotential of 0.38 V vs. SHE, which is comparable to iridium and cobalt oxide catalysts and the best among non-metal oxygen evolution electrocatalysts. Electrochemical and physical research ascribe the nitrogen/carbon materials having strong oxygen evolution activity to pyridinic-nitrogen-or/and quaternary-nitrogen related active sites (Zhao et al., 2013).

Recent studies have shown that quaternary metal chalcogenides (TMCs) can be used as electrocatalysts in hydrogen evolution reactions (HER). These catalysts have the potential to outperform their binary or ternary counterparts in electrocatalysis due to their electronically tunable structures. Maheskumar et al. developed non-precious ternary copper tin sulfide (CTS) electrocatalysts for HER, with Cu_2_SnS_3_ and Cu_4_SnS4 demonstrating promising performance (Maheskumar et al., 2018a). Bahareh et al. synthesized an electrodeposited CoNi_2_Se_4_ electrocatalyst for HER with a stable mesoporous spinel ZnFe_2_O_4_, exhibiting a large surface area and small particle size (Amin et al., 2017). Mingyue et al. reported stable mesoporous spinel ZnFe_2_O_4_ as a proficient electrocatalyst for HER, with a large surface area and small particle size (Sun et al., 2016). The mesoporous structure of the ZnFe_2_O_4_ catalyst sustained up to 700°C after stabilization treatment. The electrocatalytic activity of mesoporous ZnFe_2_O_4_-700 demonstrated an overpotential of 170 mV at a current density of 10 mA/cm^2^, with a Tafel slope of 84 mV/dec and a charge transfer resistance value (R_ct_) of 90.15 Ω, demonstrating durability for 24 h. Priya et al. reported that quaternary Cu_2_ZnSnS_4_ (CZTS), a low-cost photovoltaic material, showed promising performance in photocatalytic water splitting for hydrogen production (Kush et al., 2013). However, its electrochemical behavior in the HER is still largely unexplored. The HER polarization curve of quaternary CZTS showed a current density of 15.9 mA/cm^2^ in a 0.5 M H_2_SO_4_ electrolyte concentration and 9.8 mA/cm^2^ in 1 M H_2_SO_4_. Stability tests showed consistent performance even after 100 cyclic voltammetry (CV) cycles, indicating a faster reaction rate and superior electrocatalytic activity. This suggests that quaternary CZTS could be a promising alternative for electrocatalytic HER applications.

## 5 Evolving development of novel CZTS electrocatalyst towards water splitting reactions

The availability of energy has grown increasingly vital for human beings’ daily lives as society has advanced at a rapid pace. People’s lifestyles necessitate a large amount of energy because transportation, daily conveniences, and assets would be rendered colorless without it. Hence, ensuring energy security is paramount for the sustainable long-term growth of society; it is a facet that must not overlooked. Coal, natural gas, petroleum, nuclear and renewable energy sources are the five major categories of energy sources. Among those, fossil fuels include coal, natural gas, and petroleum, while renewables include wind, biofuels, wood, geothermal, solar energy, hydrogen power, and so on. By combining hydroelectric hydrogen and carbon dioxide emissions from metals businesses, the national energy situation is examined, as well as the creation of hydrogen-based fuels for the transportation and rising sectors in Iceland. Finally, the Icelandic Hydrogen and Fuel Cell Company is addressed, which includes five phases of a progressive transition of Iceland into a hydrogen economy in a European research endeavor. Each of the energy sources have its own set of limitations and cons. Although, fossil fuels are the primary source of energy, their rapid depletion has resulted in major issues such as greenhouse effect and contamination, particularly air pollution. Nuclear energy has the potential to produce radioactive fission, which is hazardous to human health. Furthermore, solar energies are constrained by geography and big areas. In comparison to fossil fuels and nuclear energy, most renewable energies offer various advantages, including purity, abundance, and environmental friendliness. Therefore, Hydrogen energy, in particular, is the most ecologically benign and has the highest energy density. As a result, sustainable hydrogen generation is becoming increasingly crucial in addressing the energy crisis and environmental challenges, and it is receiving a lot of attention. Electrochemical water splitting is a promising approach to turn electrical energy into chemical energy for energy needs and as a result, H_2_ fuel can be used as a non-intermittent sustainable energy supply. HER $\left.{\left(2H\right.}^{+}{+2e}^{‐}{\;\to \;H}_{2}\right)$ at the cathode and OER $\left({2H}_{2}{O\;\to \;O}_{2}{+4H}^{+}{+4e}^{‐}\right)$ at the anode are the two half-reactions that overall makeup the water splitting.

**FIGURE 2:**
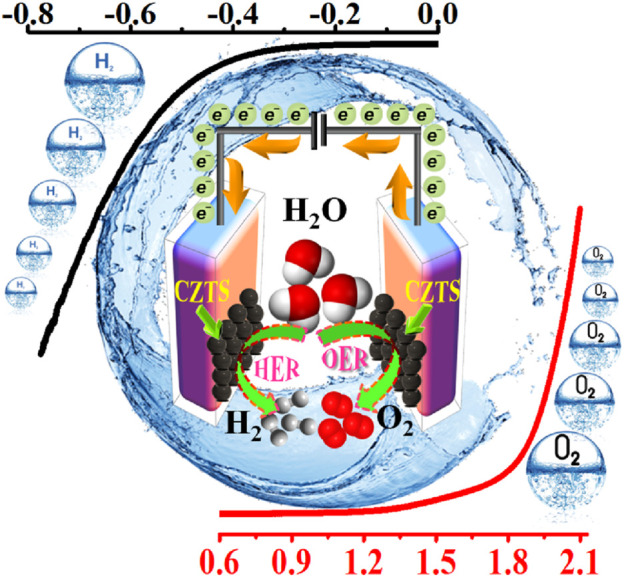
Schematic illustration of water splitting (both HER and OER) on CZTS based electrocatalytic systems.

With low overpotential and Tafel slope, Pt group metals and Ru/Ir-based compounds are presently the most sophisticated, noble, and efficient HER and OER electrocatalysts (Zhu et al., 2015). However, the expensive cost of these noble electrocatalysts as well as their rarity, limit their use in producing H_2_ resources through water splitting. Since H_2_ gas (a pure chemical fuel) may be a better answer for environmental emissions, sustainability, and energy security concerns (Liu et al., 2016), it is a very uncommon natural gas on the planet. As a result, a water electrolyzer employing an earth-abundant (and hence cheaper) efficient electrocatalyst can be utilized to produce H_2_ gas at a low cost and efficiency, making it a viable alternative to a water electrolyzer using a scarce expensive noble electrocatalyst. As a result, developing efficient, earth-abundant, low-cost, low voltage, and stable electrocatalysts for water splitting (HER and OER) is critical (Zhang et al., 2015). For HER and OER, various types of earth abundant electrocatalyst are available. As a result, producing effective cheaper earth abundant HER and OER bifunctional electrocatalysts can greatly boost total water splitting efficiency. The literature signifies, CZTS is one of the finest earth-abundant electrocatalysts for water splitting processes, with lower overpotential (335 mV), a lower charge transfer resistance (6Ω), and a greater exchange current density (882 mA/cm^2^). The findings obtained are superior to those obtained by previously published chalcogenide-based systems (Kush et al., 2015). Furthermore, even after 300 continuous CV cycles, current density loss is nearly non-existent, confirming CZTS NPs are having exceptional stability and good catalytic activity. Furthermore, CZTS has a 1.86 V overpotential, a charge transfer resistance of 178, and a lower Tafel slope of 144 mV/dec towards OER. It is anticipated that our work will contribute to the creation of novel and efficient bifunctional electrocatalysts based on transition metal chalcogenides Figure 2. Shows the schematic illustration of water splitting on CZTS based electrocatalytic systems. Several approaches for the production of CZTS nanoparticles, such as sol-gel (Kumar et al., 2016), solvothermal (Gong et al., 2016), mechanochemical (Pareek et al., 2015), spray pyrolysis (Courel et al., 2017), spin coating (Safdar et al., 2016), chemical vapor deposition, hybrid sputtering, and pulsed laser deposition have been published in order to develop low-cost, high-efficiency solar cells (Vanalakar et al., 2015). Tables 1, 2 shows the comparison of the electrocatalytic HER and OER performance of chalcogenides based electrocatalysts with noble metals.

**TABLE 1:** Comparison of the electrocatalytic HER performance of chalcogenides based electrocatalysts with noble metals.

Sr. No.	Materials	η (mV vs. RHE)	Tafel slope (mV/dec)	Ref.
1	F-MoS_2_	380	175	Guo et al. (2016)
2	Bulk WS_2_	290	119	Zhao et al. (2016)
3	MWCNTs@Cu	366	109	Li et al. (2015a)
4	MWCNTs@MoS_2_	148	84	
5	MWCNTs@Cu@MoS_2_	146	62	
6	MoP_2_/Mo	273	69	Pu et al. (2016)
7	Cu−MoS_2_/rGO	−126	90	Li et al. (2015b)
9	Co_9_S_8_	224	135	Pan et al. (2015)
11	CZTS	300	85	Digraskar et al. (2017)
12	Co-CZTS	200	73	Digraskar et al. (2018)
14	Cu_2_SnS_3_	330	98	Maheskumar et al. (2018a)
15	Cu_4_SnS_4_	358	110	
16	MoS_2_ NS	280	90	Muralikrishna et al. (2015)
17	Cu_3_SnS_4_	230	76	Maheskumar et al. (2018b)
18	Cu_3_SnS_4_	250	76	Govindaraju et al. (2021)
19	Cu_3_SnS_4_-rGO	190	64	
20	CZTS NCs with 1.0 OM	561	157.6	Li et al. (2023)
21	NiS	560	182	Hod et al. (2015)
22	SnS-N-doped graphene	-	266	Shinde et al. (2015)
23	MoS_2_/SnS_2_	288	50	Xiao et al. (2020)
24	SnS_2_-Pt-3	210	50	Yu et al. (2020)
25	CZTS-PEG	295	133	Hu et al. (2024)
26	CuFeS_2_	−88.7	47	Li et al. (2017)
27	Cu_2_WS_4_	−650	121	Tiwari et al. (2018)
28	Cu_2_MoS_4_	−333	130.3	Kim et al. (2017)
29	Cu_2_MoS_4_/MoSe_2_ nanostructures	−166	74.7	Tiwari et al. (2018)
30	Cu_2_ZnSnS_4_	∼−1,200	52	Kush et al. (2015)
31	NiCuCoS_3_-modified GE	−600	116.2	Asiri et al. (2021)
32	MoS_2_	−610	∼200	Chua et al. (2016)
33	Mo_0.93_Sn_0.07_S_2_	−403	170	Radhakrishnan et al. (2022b)
34	MoS_2_/NiO/MWCNT		289	Lai et al. (2019)
35	CuGaS_2_/7 wt %MoS_2_	464	56.2	Radhakrishnan et al. (2022a)
36	Mn-MoS_2_/rGO	110	76	Wu et al. (2017)
37	MoSe_2_-NiSe	210	56	Liang et al. (2015)
38	MoS_2_/rGO	141	136	Digraskar et al. (2018)
39	MoS_2_ NFs/rGO	190	95	Ma et al. (2014)
40	3D MoS_2_/rGO	125	41	Zhang et al. (2015a)
41	CZTS/MoS_2_-rGO	50	68	Digraskar et al. (2021)
42	OAm-GO/CZTS	47	64	Digraskar et al. (2019c)

**TABLE 2:** Comparison of the electrocatalytic OER performance of chalcogenides based electrocatalysts with noble metals.

Sr. No.	Materials	η (mV vs. RHE)	Tafel slope (mV/dec)	Ref.
1	AgCuZn sulfide	39	95	Xie et al. (2015)
2	CuS/SnS_2_/rGO	264	67	Yao et al. (2021)
3	Ag_2_BaSnS_4_	300	70	Jabeen et al. (2023)
5	CuCo_2_S_4_	310	86	Chauhan et al. (2017)
6	Cu2ZnSnS_4_	382	105	Pham et al. (2017)
7	Ag_2_ZnTiS_4_	275	43	Aslam et al. (2023)
8	CoNi_2_Se_4_	1.61V vs. RHE	--	Amin et al. (2017)
9	Co_0.13_Ni_0.87_Se_2_/Ti	1.62 V vs. RHE	94	Liu et al. (2016b)
10	CoSe	1.5 V vs. RHE	69	Liu et al. (2015)
11	Ni-Co-S/CF	1.67 V vs. RHE	109	Hu et al. (2016)
12	OAm-GO/CZTS	1.11 V vs. RHE	91	Digraskar et al. (2019b)

Controlled synthesis procedures are used to create nanocrystals with exact composition, phase, surface structure, and dispersibility in appropriate solvents in order to make high-quality CZTSSe films. Three primary methods have been established for the production of the CZTSSe absorber layer:

### 5.1 Synthesis of colloids

Using thermolysis procedures, this approach creates colloidal quaternary CZTS or CZTSe nanocrystals. To avoid aggregation and guarantee dispersibility in nonpolar solvents, these nanocrystals are coated with organic ligands (such as oleylamine) over an inorganic core with a specified composition and crystal phase.

### 5.2 Binary/ternary nanocrystals

By mixing various nanocrystals, CZTSSe films may be made from binary or ternary colloidal nanocrystals, providing flexibility in modifying the absorber layer’s composition and characteristics.

### 5.3 Hydrophilic CZTS nanocrystals

To create hydrophilic nanoparticles, CZTS nanocrystals may be generated using solvothermal techniques and then dispersed in polar solvents with the use of ligands such as polyvinylpyrrolidone (PVP) and ethylene glycol. However, because film formation presents difficulties, devices based on hydrophilic CZTS nanocrystals frequently perform less well. Solar cells utilizing colloidal CZTS nanocrystals have exhibited encouraging results, as evidenced by power conversion efficiency values of 9.85 for quaternary CZTS and 8.5 18 for the binary ZnS and ternary Cu_2_SnS_3_ combination (Cao et al., 2012). The synthesis of hydrophobic CZTS, CZTSe, and CZTGeS nanocrystals entails regulating the reaction products size, shape, content, and phase. Burst nucleation of molecular precursors in coordinating solvents is used to create these nanocrystals, which are then grown in a controlled manner. A broad range of semiconductor nanocrystals, such as II-VI, III-V, I-VI, IV-VI, and group IV materials, are synthesized by the colloidal chemistry approach. This process may also be used to produce ternary and quaternary compounds such as CuInS_2_, CuInSe_2_, CuGaSe_2_, CIGS, and CZTS. All things considered, the production of hydrophobic and hydrophilic CZTS nanocrystals is essential to the development of high-performing CZTSSe-based solar cells (Yin and Alivisatos, 2005).

#### 5.3.1 CZTS nanocrystals

Colloidal CZTS nanocrystals are synthesized by dissolving copper (Cu), zinc (Zn), and tin (Sn) compounds in long-chain coordinating solvents, then mixing them with a sulfur source and heating the solution to a specific temperature (Guo et al., 2010). This process leads to burst precipitation and controlled growth, yielding quaternary compound semiconductor nanocrystals with a kesterite crystal structure of high phase purity. The phase of the CZTS nanocrystals can be varied, ranging from kesterite or stannite phases with a tetragonal unit cell to the wurtzite phase with a hexagonal crystal cell. Achieving the kesterite phase, which is favorable for photovoltaic applications, requires careful control of the chemical environment during synthesis, including the coordination strengths and reactivity between ligands and cations in the solution. Oleylamine is commonly used as a solvent due to its high boiling point and coordinating ability with chalcogenide compounds. Other compounds like trioctylphosphine oxide (TOPO), oleic acid (OA), and octadecene (ODE) are also occasionally used (Steinhagen et al., 2009). Sulfur can be incorporated into the reaction system by dissolving elemental sulfur directly into oleylamine, or other sulfur sources like dodacanethiol (DT) can be utilized to provide the sulfur component and control crystal growth (Singh et al., 2012). The original synthesis of CZTS nanocrystals involved hot injection of a solution of elemental sulfur in oleylamine into a solution containing copper (II) acetylacetonate, zinc acetylacetonate, and tin (IV) bis(acetylacetonate) dibromide in oleylamine at 225°C (Guo et al., 2009). The resulting CZTS nanocrystals exhibit mild polydispersity, with most falling in the 15–25 nm range. High-quality CZTS nanocrystals were achieved by adjusting the stoichiometric metal precursor ratio to be Zn-rich and Sn-poor, producing a photovoltaic efficiency of 7.2% in 2010 (Guo et al., 2010). Subsequent progress has led to various other recipes for forming high-quality crystals, with different precursor types, reaction temperatures, times, precursor injection modes, and routes for incorporating sulfur into the reaction (Chesman et al., 2013). However, the performance from synthesized nanocrystals is rarely discussed in reported results.

#### 5.3.2 CZTSe nanocrystals

CZTSe nanocrystals are synthesized by introducing selenium sources to precipitate from metallic precursors in coordinating solvents, resulting in quaternary compound semiconductors with a kesterite crystal structure and high phase purity. Metal salts commonly used for synthesizing CZTSe nanocrystals include chlorides, acetates, and acetylacetonates, similar to those used for CZTS nanocrystals (Liu et al., 2012). However, selenium-based nanocrystal synthesis typically involves soluble selenium precursors in alkyl phosphines like trioctylphosphine (TOP) and tributyl phosphine (Shavel et al., 2010). A phosphine-free route for synthesizing high-quality CZTSe nanocrystals in organic solvents has been developed. This method involves reducing selenium powder with dodacanethiol (DT) in the presence of oleylamine (OM) at room temperature to generate a soluble alkylammonium selenide. This approach produces CZTSe nanocrystals with small size, excellent monodispersity, and strong absorbance in the visible region (Liu et al., 2012). However, the power conversion efficiency (PCE) using CZTSe nanocrystals is currently less than 5%, significantly lower than devices based on CZTS nanocrystals. This lower efficiency is attributed to less effective selenization, resulting in an inferior grain structure for the CZTSe film. Despite this, CZTSe nanocrystals offer precise control of the final CZTSSe films with tunable band gaps, or even gradient band gaps, to optimize device performance further.

#### 5.3.3 CZTGeS nanocrystals

CZTGeS nanocrystals are synthesized similarly to CZTS, with germanium partially substituting tin. Germanium chloride is commonly used as the germanium precursor, along with copper acetylacetonate, zinc acetylacetonate hydrate, tin bis(acetylacetonate) dibromide, and sulfur dissolved in oleylamine. These CZTGeS nanocrystals are polydisperse, with particle sizes ranging from 5 to 15 nm. Compared to CZTS nanocrystals, the diffraction peaks of CZTGeS shift systematically toward higher 2θ angles, indicating the contraction of the tetragonal unit cell due to the smaller germanium atoms replacing tin. CZTGeS nanocrystals have been utilized to fabricate CZTGeSSe thin films, which have demonstrated impressive device performance values as high as 8.4% (Ford et al., 2011).

#### 5.3.4 Synthetic chemistry for size, shape, composition, and phase control

CZTS and CZTSe nanocrystals are selected as the typical examples to explain the general synthetic chemistry for size, shape, composition and phase control. Other analogous nanocrystals, e.g., CZTSSe nanocrystals and CZTGeS nanocrystals, share a similar reaction mechanism.

##### 5.3.4.1 Size control

Controlling the size of CZTSSe nanocrystals is essential for creating high-performance films. It is ideal to keep the average particle size under a few tens of nanometres to ensure good solubility in the desired solvent. This size range is typically achieved by adjusting the reactivity of precursors and the coordinating ability of surfactants and solvents. In most successful cases, the average particle size falls between 10 and 40 nm. Longer reaction durations and slower reaction rates of chalcogenide precursors tend to produce larger nanocrystals. However, in some cases, much smaller CZTS nanocrystals with diameters between 2 and 7 nm can be controllably achieved by an accelerated decomposition reaction. While controlling the size of semiconducting nanoparticles can alter their optical properties due to quantum confinement, the effects of quantum confinement become negligible in these devices since they are based on annealed CZTSSe films, where nanoparticles combine into large grains (Rath et al., 2012).

Achieving a very narrow size distribution of the initial CZTS nanoparticles is necessary to obtain a close compact film, as the attractions between semiconductor nanoparticles are weak. Further study on the size effect could be carried out by fixing other parameters, such as composition and the selenization process, to demonstrate their effects on device performance. The particle size, which affects the specific surface area of the nanocrystals, can influence the number of surface ligands present in as-deposited films, which in turn can further influence grain growth and device performance. The trade-off between impurities left behind by decomposing surface ligands and the need for high dispersibility in the precursor inks remains an ongoing dilemma in nanoparticle-based solar cell fabrication.

##### 5.3.4.2 Compositional control

Controlling the composition of metal cations is crucial for producing high-performance CZTSSe films due to the narrow phase stability and volatile nature of some elemental constituents at high temperatures. The chemical composition of CZTS or CZTSe nanocrystals can be adjusted by varying reaction conditions such as temperature, type of precursor, and reagent concentration. One challenge in compositional control arises from the precipitation of Cu_2_S, which has a small solubility product, before the formation of CZTS. The transformation of Cu_2_S to CZTS is facilitated by the high atomic diffusivity of copper. Among the binary chalcogenides involved (Cu_2_S, ZnS, and SnS_2_), ZnS has the largest solubility product and is the last constituent to be incorporated into the CZTS phase. This often leads to a lower zinc content in the final nanocrystal composition compared to the initial precursor solution (Wei et al., 2010). Achieving a highly predictable composition requires balancing the reactivity of metal precursors and the chalcogenide source, as well as controlling reaction conditions such as time and temperature. These factors are essential for developing synthetic schemes capable of producing quaternary compounds with the desired composition.

##### 5.3.4.3 Morphology control

The morphology of CZTS or CZTSe nanocrystals is significantly influenced by the selection of surfactants and reaction conditions. Various surfactants can selectively bind to specific facets, enabling anisotropic growth in certain directions. For instance, dodacanethiol and tert-dodecyl mercaptan can bind to facets other than (002), resulting in the formation of CZTS nanorods (Singh et al., 2012). Moreover, changes in reaction time can lead to different morphologies, ranging from sphere-like to rhombus-like and rice-like shapes, as the reaction time is extended (Li et al., 2012). While controlling the morphology in CZTS nanoparticle synthesis primarily impacts the formation of ideal building blocks for film formation rather than directly affecting the electrical properties of the final materials, it plays a crucial role in the high-temperature annealing process during device fabrication.

##### 5.3.4.4 Phase control

The synthesis of CZTS nanocrystals with a pure kesterite phase and minimal secondary phases like Cu_2_SnS_3_ and ZnS is critical due to the narrow phase window. In colloidal nanocrystal synthesis, the choice of capping ligands and the reactivity of the precursors strongly influence the crystallographic phase of the nanocrystals. Kesterite is the preferred phase due to its thermodynamic stability. Precursors with lower reactivity and solvents or surfactants with weaker coordination toward metal cations tend to favour the formation of the kesterite phase (Lu et al., 2011). For instance, oleic acid promotes the kesterite CZTS structure, while alkanethiols facilitate wurtzite CZTS formation due to stronger metal cation-thiol group coordination, encouraging hexagonal structure formation. Presently, all functional devices are based on kesterite CZTS nanocrystals, expected to continue demonstrating superior device performance compared to wurtzite-based ones. However, wurtzite nanocrystals could still be a viable route for ink fabrication if they are easier to synthesize or formulate into inks, as the metastable wurtzite structure can transform into the more stable kesterite structure after high-temperature annealing.

### 5.4 CZTSSe phases based on colloidal nanocrystals and MCC ligands

The solution deposition of CZTSSe films involves using colloidal Cu_2−x_Se and ZnS nanocrystals (NCs) capped with long-chain alkylamine ligands, treated with (NH_4_)_4_Sn_2_S_6_^4−^ metal chalcogenide complexes (MCCs). These complexes replace the original ligands, forming stable colloidal solutions. The solutions are combined, deposited, and annealed to form highly crystalline CZTSSe films. This approach reduces carbon residue after annealing, as inorganic ligands replace alkylamine ligands. Another method uses Cu_2−x_S NCs and Zn/Sn ligands with a simple reaction pathway, showing promise for reducing carbon residues. Various successful methods exist for synthesizing CZTS nanocrystals, including solvothermal methods. One modified solvothermal method uses ethylene glycol containing PVP and Na_2_S·9H_2_O to synthesize CZTS. PVP acts as capping ligands, making the CZTS nanocrystals hydrophilic and stable in ethanol and water. Novel structures like hierarchical CZTS particles and CZTSe nanosheets can be achieved, showing promise for carrier transport and collection.

While the thermolysis method offers fine control over nanocrystal properties, solvothermal growth provides a variety of metastable structures. These methods, not reliant on long-chain ligands, reduce undesirable carbon residue but may suffer from inferior solubility. Despite challenges, ongoing advancements in synthesis methods enhance CZTSSe film formation and device performance.

### 5.5 Doped CZTS based HER

**FIGURE 3:**
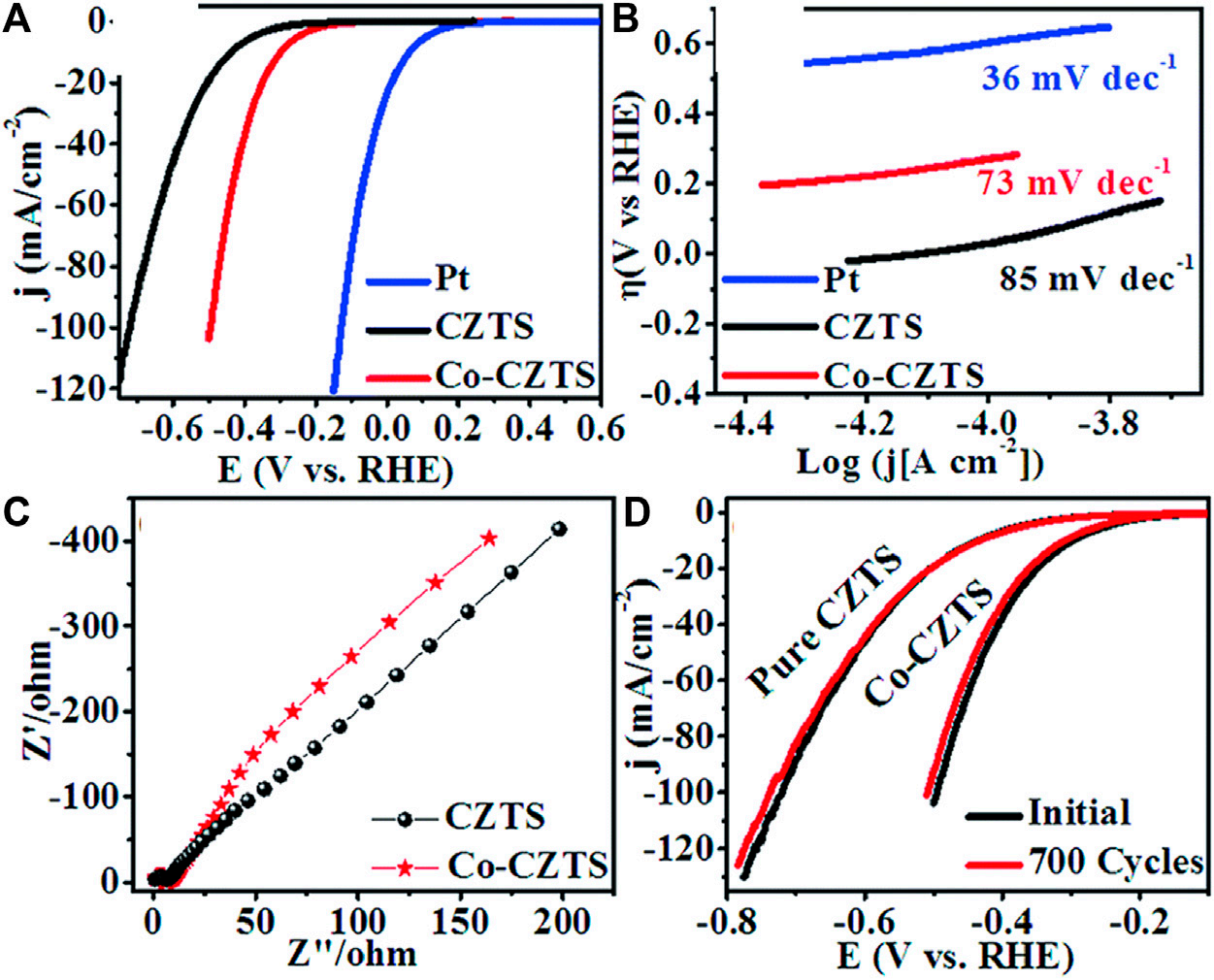
Superimposed images of **(A)** LSV curves for HER, **(B)** corresponding Tafel plots, **(C)** Nyquist plots of the CZTS and Co-doped CZTS NPs, and **(D)** Durability test of Co-CZTS in 0.5 M H_2_SO_4_.

Literature reflects, one of the greatest ways is to improve the catalytic performance of electrocatalysts by doping them with heteroatoms or transition metals/metal oxide. The influence of doping on the catalytic performance of Co-CZTS hydrogen evolutionary hybrid catalysts, for example, is uncommon. Based on the results of the preceding study and HER-related investigations, Renuka, et al. developed a noble metal-free Co-doped CZTS catalyst for hydrogen evolution reaction (HER) using a sonochemical technique. The optimized Co-doped CZTS electrocatalyst demonstrates exceptional HER performance as shown in Figures 3A–D with a minimal overpotential of 298 mV at 10 mA/cm^2^ leading to a Tafel slope of 73 mV/dec with small charge transfer resistance (Rct = 4Ω), as well as high stability up to 700 cycles with little cathodic current loss. The co-doped CZTS-based HER system ease of synthesis and high activity not only provides a low-cost, high-activity HER system, but it also opens a new avenue for investigating the use of metal doping in chalcogenides as an attractive catalyst for electrocatalytic applications in energy conversion devices (Digraskar et al., 2018).

**FIGURE 4:**
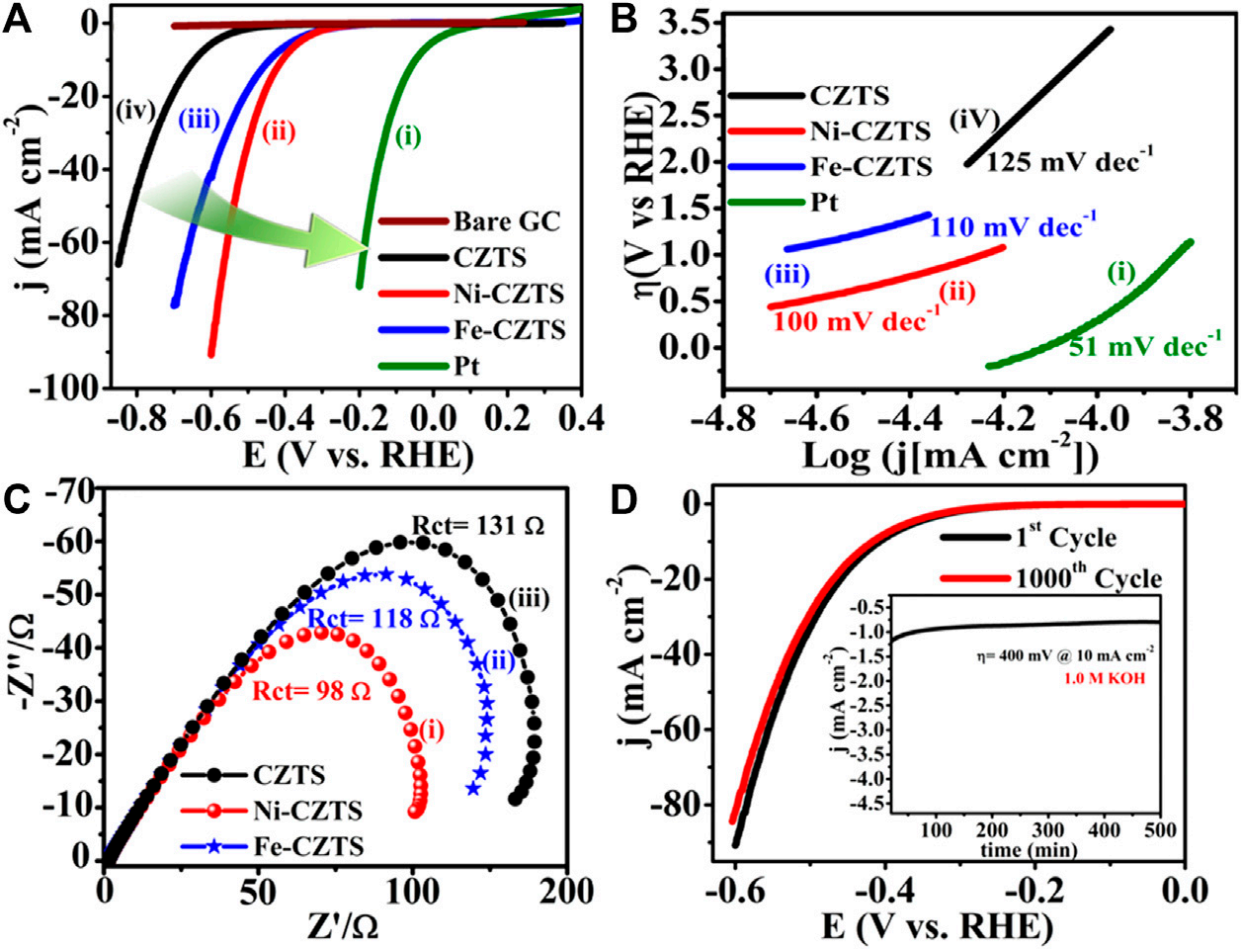
**(A)** Superimposed HER polarization curves showing the high catalytic activity of Ni-CZTS, **(B)** corresponding Tafel plots, **(C)** Nyquist plot of Ni-CZTS, **(D)** The durability test of Ni-CZTS in 0.5 M H_2_SO_4_.

**FIGURE 5:**
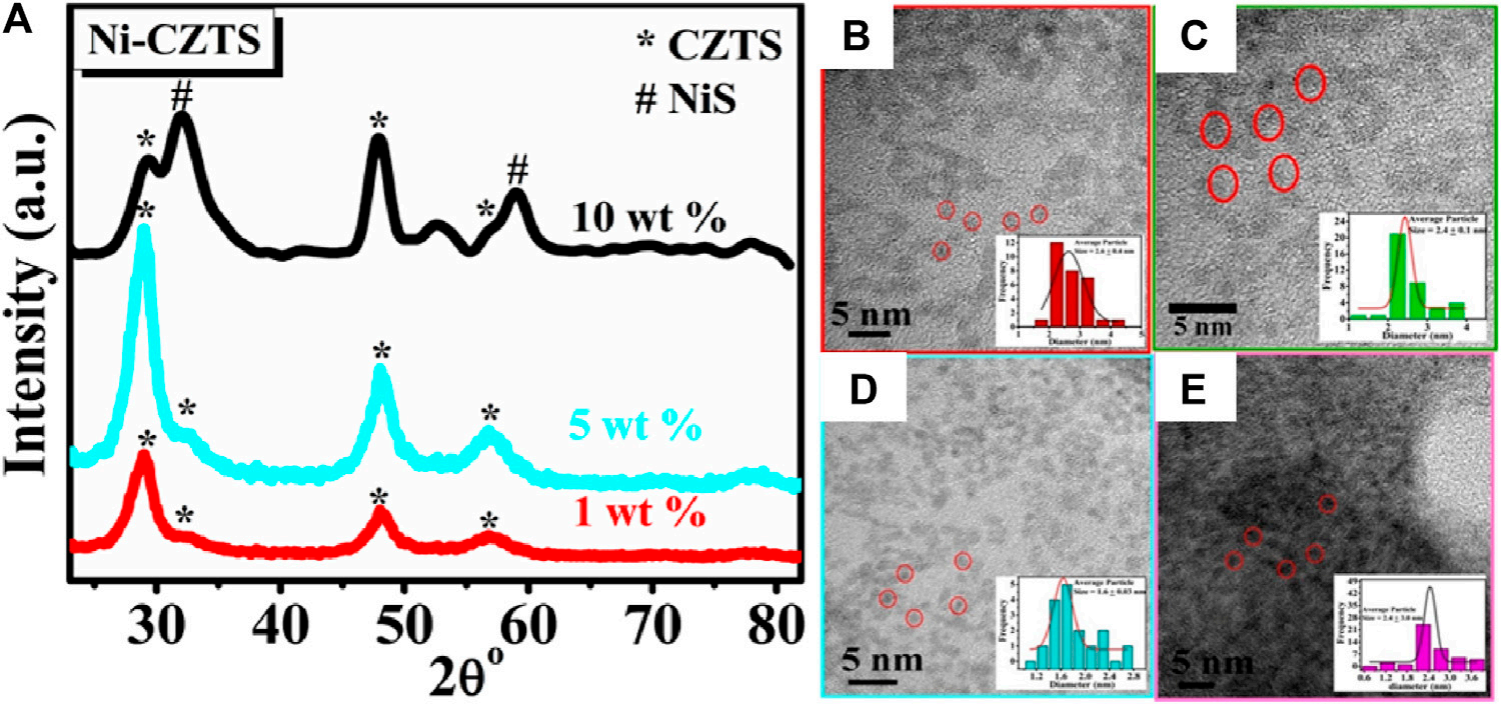
**(A)** X-ray Diffraction studies towards the comparison of Ni doping in CZTS with different Wt %, **(B–E)** HR-TEM images of **(B)** pure CZTS NPs (inset shows particle size distribution plots of CZTS with particle size 2.6 ± 0.4 nm); **(C)** Co-doped CZTS NPs (inset shows particle size distribution plots Co-CZTS with particle size 2.4 ± 0.1 nm); **(D)** Ni-doped CZTS NPs (inset shows particle size distribution plots Ni-CZTS with particle size 1.6 ± 0.03 nm); and **(E)** Fe-doped CZTS NPs (inset shows particle size distribution plots Fe-CZTS with particle size 2.4 ± 3.0 nm).

Renuka et al. present the use of sonochemical synthesis of nickel and iron (M = Ni, Fe) doped Cu_2_ZnSnS_4_ (CZTS) as an electrode for improved electrocatalytic water splitting performance. The as-prepared electrode materials were further characterized by Raman, X-ray photoelectron (XPS), transmission electron microscopy (TEM), and X-ray diffraction (XRD) spectroscopic investigations. Significantly, the HER in 0.5 M H_2_SO_4_ and 1 M KOH electrolyte solutions has a low overpotential of roughly 214 and 400 mV, respectively. Small Tafel slopes and long-term durability tests, i.e., up to 500 min for water splitting, shows that Ni-doped CZTS is an effective bifunctional electrocatalyst with high electrocatalytic activity and exceptional stability. Furthermore, an overpotential for the acidic and alkaline HER are 300 mV in 0.5 M H_2_SO_4_ and 445 mV in 1.0 M KOH respectively. This high-efficiency can address the current energy shortage. The bifunctional electrocatalyst Ni-doped CZTS shows the HER in 0.5 M H_2_SO_4_ reaches the current density of 10 mA/cm^2^ and has a low overpotential of around 214 mV vs. RHE. The Ni-doped CZTS Tafel slope (80 mV/dec) is lower than pure CZTS (85 mV/dec). By comparing Ni-CZTS activity and taking into consideration the higher Ni loading in CZTS. The activity of the catalyst is shown to diminish as the Ni loading increases (Figure 4). This might be owing to the excess addition of Ni totally altering the internal arrangement of CZTS and causing a change in its crystal structure, as evidenced in XRD tests showed in Figure 5A). Figures 5B–E, illustrates the electrocatalytic performance of Ni, Fe, and Co-doped CZTS NPs. Interestingly, the morphological characteristics of HER activity have been observed as Ni-CZTS > Co-CZTS > Fe-CZTS > CZTS. Furthermore, the polarization curve after 1000th cycles of continuous CV scanning reveal no modification in characteristics from the beginning, demonstrating that electrocatalysts are quite stable. Furthermore, the Fe-doped CZTS electrocatalyst has a weak response to HER, with an overpotential of 300 mV in 0.5 M H_2_SO_4_ and a Tafel slope of 82 mV/dec (Digraskar et al., 2019a).

Sheebha et al. previously demonstrated the synthesis of CXTS (X = Zn, Co., Mn, Ni) through the hydrothermal method for applications in hydrogen evolution reaction (HER) and dye degradation (Sheebha et al., 2020). Their findings highlighted CNTS as possessing high HER activity, while CZTS emerged as a promising candidate for dye degradation. Inspired by their efforts, Prashant et al., started developing Cu2CoSnS4 (CCTS) for HER applications. This involved substituting Zn^+2^ with Co.^+2^ in CZTS, resulting in a reduction of antisite defects and cation disorder (Ghediya et al., 2019). CCTS, characterized as an earth-rich, environmentally friendly, and cost-effective material, shares key properties with CXTS, such as analog configuration, tunable bandgap, and low resistivity.

These attributes make CCTS suitable for applications in optoelectronics, energy storage, and energy conversion devices (Krishnaiah et al., 2019). To streamline the synthesis process, employed a direct deposition approach on conducting substrates, eliminating the need for high temperatures or vacuum conditions. A significant benefit for electrochemical hydrogen evolution is the absence of surfactants and binders in the large-area electrodes that are produced, as these substances have the potential to break down in lengthy processes. A silver substrate was immediately covered with the precursor ink, which greatly aided in the good adhesion of the material development. However, agglomeration and voids on the silver electrode caused a non-uniform distribution, as seen by scanning electron microscopy (SEM). Despite this, the CCTS electrode demonstrated outstanding performance in HER by electro catalyzing the splitting of water. After around 18 hours of stability testing, the electrode showed a low overpotential of −125.7 mV vs. RHE at −10 mA/cm^2^, which rose slightly to −164.3 mV vs. RHE at −10 mA/cm^2^. Enhanced electron transport was suggested by the modest Tafel slope of 115.7 mV/dec, which later decreased to 101.7 mV/dec. Moreover, the CCTS electrode displayed notable stability under an applied current of −10 mA/cm^2^ for approximately 18 h, reinforcing its potential for practical applications in hydrogen evolution (Joshi et al., 2022).

### 5.6 Carbon nanostructured composites of CZTS for HER

**FIGURE 6:**
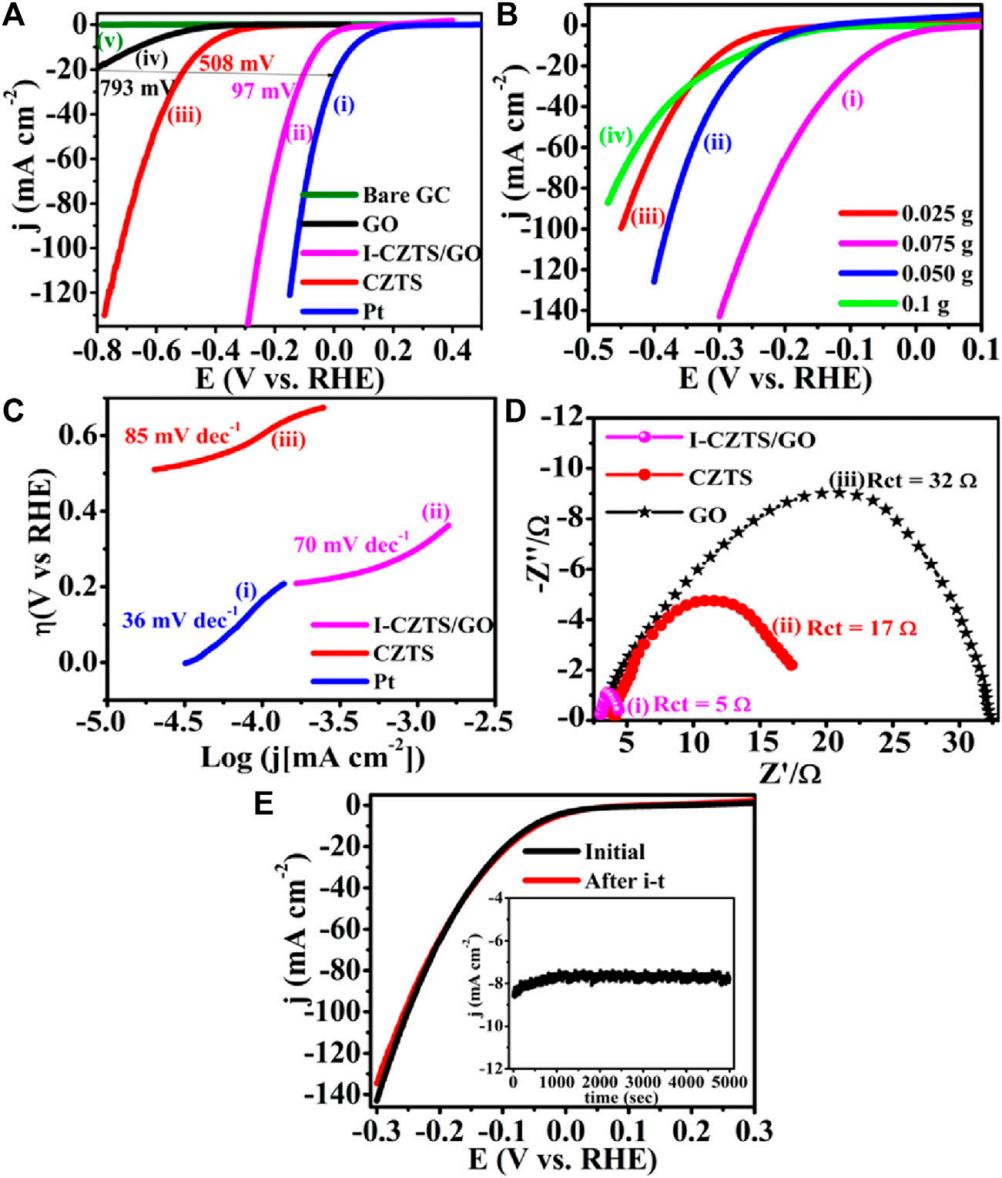
**(A)** Superimposed polarization curves for HER; **(B)** LSV of I-CZTS/GO composite GO with different loading; **(C)** corresponding to the Tafel plots; **(D)** Nyquist plots; and **(E)** The durability test of I-CZTS/GO using SCE and Pt wire as reference and counter electrodes in 0.5 M H_2_SO_4_ at scan rate 50 mV/s.

Recent research on carbon-based catalysts such as graphene oxide (GO) and carbon nanotubes (CNT) has revealed that their unique physicochemical features make them suitable substrates for improving electrocatalytic activity (Pan et al., 2015). The pristine carbon materials are electrochemically inert or possess poor catalytic activity. Due to their large surface area and strong electron conductivity, the use of carbon-based materials as the support improves the conductivity of hybrid catalysts and increases the dispersity of the active components. As a result, the catalytic activity of carbon-based composite catalysts is frequently increased. Graphene, a few layers, and two-dimensional graphene materials have gotten a lot of attention as HER catalyst support materials as of large surface area and efficiency, graphene sheets as a support material improve the conductivity of hybrid catalysts and increase the dispersion of active components Figure 6A.

The overpotential of the I-CZTS/GO composite is 39.3 mV vs. SHE at 2 mA/cm^2^, with overpotentials of 53.1 and 97 mV vs. SHE to allow current densities of 10 and 20 mA/cm^2^, respectively. This modest overpotential corresponds to the greatest catalytic activity of the previously reported HER catalysts, implying that pure CZTS and bare GO electrodes have lowered HER activities than I-CZTS/GO composite catalysts. The electrical and chemical coupling of CZTS NPs and GO supporters is responsible for the increased electrochemical performance of CZTS/GO composites (Ma et al., 2014; Niyitanga and Jeong, 2017). Figure 6B shows the comparison I-CZTS/GO composite by using a different amount of GO loading, i.e., 0.025, 0.050, 0.075, and 0.1 g. CZTS/GO NCs with 0.075 g of GO loading, on the other hand, produce superior results than all other ratios, hence it is used in all later studies. The main parameter used to evaluate HER effectiveness is the Tafel slope; a lower Tafel slope denotes a faster rate of increase in HER rate as overpotential increases. Figure 6C illustrates the Tafel slope of I-CZTS/GO, which is 70 mV/dec. On the one hand, this Tafel slope is larger than Pt 36 mV/dec, yet it is lower than pure CZTS 85 mV/dec.

The electrochemical impedance spectroscopy (EIS) experiments were also performed to better understand the function of electrode kinetics and interface reaction in the HER of CZTS and its GO composites. The charge transfer resistance (*R*_*ct*_) at the electrode and electrolyte contact is responsible for the semi-circle. According to the impedance fitting, the I-CZTS/GO has a lower *R*_*ct*_ (5Ω) than the CZTS (17Ω) as depicted in the Nyquist plots of the samples Figure 6D. The better the electron transfer processes are enhanced by reducing the *R*_*ct*_ value of the I-CZTS/GO, the better the HER. In addition, I-CZTS/GO improved their catalytic performance after incorporating GO sheets, because using conducting graphene due to its exclusive electrical properties increases the conductivity of CZTS/GO and small Tafel slope of ∼70 mV/dec. The charge transfer resistance (5Ω) and exchange current density (908 mA/cm^2^) shows better electrocatalytic performance than pure CZTS. The minor reduction in catalytic activity might be due to catalyst contamination from the electrode (Popczun et al., 2013; Kush et al., 2015). As shown in the inset of Figure 6E performance for 5000th seconds of i-t amperometry for stability test (Digraskar et al., 2019c).

An attractively beautiful and optional way to address concerns about energy and greenhouse gas emissions is to utilize bifunctional electrocatalysts based on non-noble metals for the entire water-splitting process. In this work, a higher bifunctional electrocatalyst for the HER and OER called oleylamine-functionalized graphene oxide/Cu_2_ZnSnS_4_ composite (OAm-GO/CZTS), is produced and investigated. With Tafel slopes of 64 and 91 mV/dec, respectively, and overpotentials of 47 mV for HER and 1.36 V for OER at a current density of 10 mA/cm^2^, the OAm-GO/CZTS exhibits remarkable electrocatalytic activity and endurance toward H_2_ and O_2_ in both basic and acidic environments. These findings imply that adding electron-donating functional groups to catalysts might considerably boost catalytic activity, offering a new path toward developing extremely efficient water splitting catalysts that could eventually replace noble metal-based catalysts. Oleylamine functionalized GO/CZTS composite (OAm-GO/CZTS) is prepared and explored as a superior electrocatalyst for HER.

**FIGURE 7:**
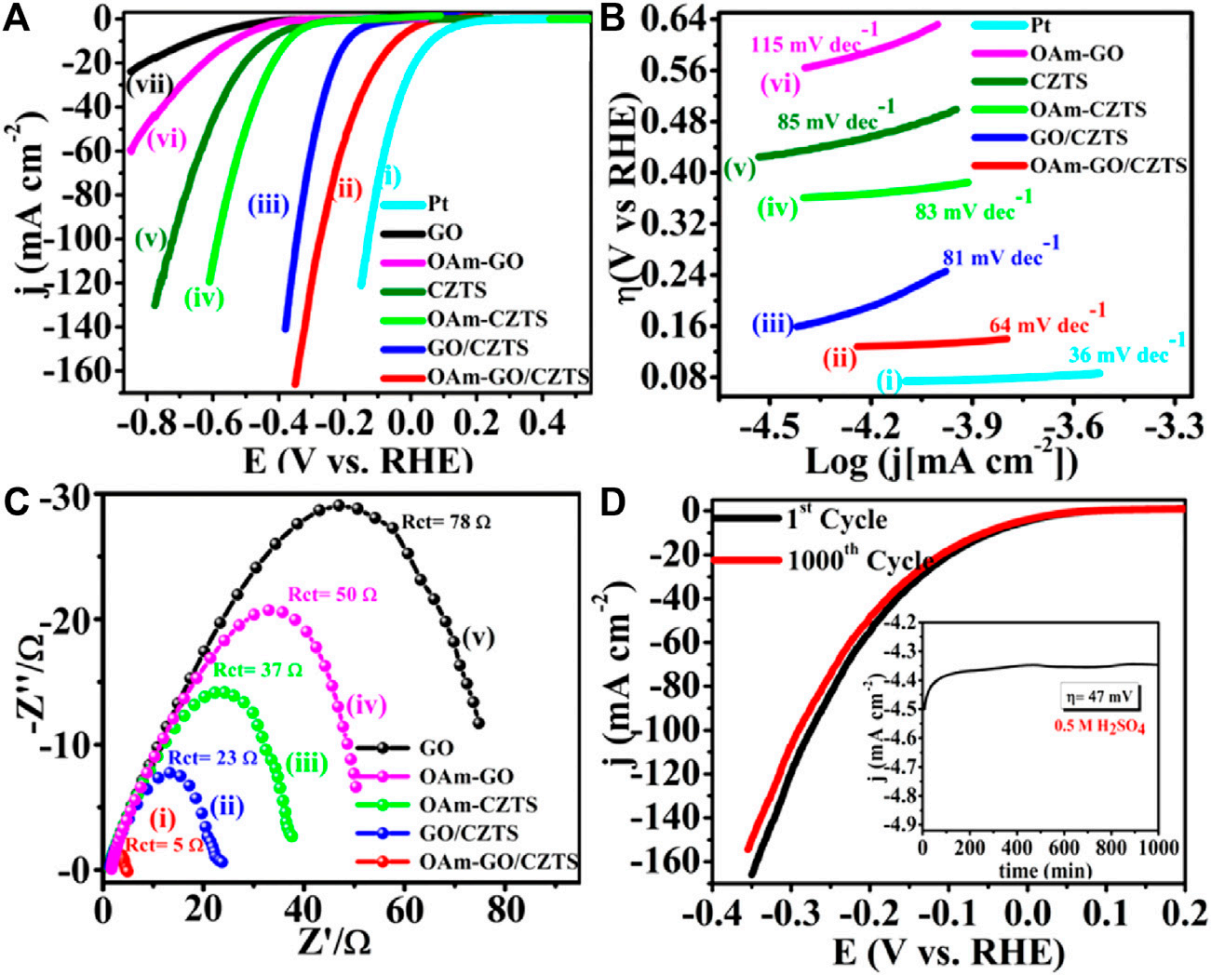
**(A)** Superimposed polarization curves for HER showing the highest catalytic activity of OAm-GO/CZTS; **(B)** corresponding Tafel plot; **(C)** Nyquist plot; **(D)** The durability test of OAm-GO/CZTS (inset of (d)) shows chronoamperometric test for 1,000 min in 0.5 M H_2_SO_4_.

In order to achieve current densities of 10 and 20 mA/cm^2^, Figure 7A OAm-GO/CZTS onset overpotentials of 47 and 96.6 mV were used. At 10 mA/cm^2^, OAm-GO (546 mV), pure CZTS (435 mV), OAm-CZTS (389 mV), and GO/CZTS composite (208 mV) electrocatalysts all had lowered HER activity than OAm-GO/CZTS. The chemical and electrical connection between CZTS NPs and OAm-GO supports, on the other hand, is responsible for the increased electrochemical performance of OAm-GO/CZTS composites. Due to its distinct electrical properties, researchers think that the amine group is a major factor in the increased electrocatalytic activity and that graphene might be a very useful conductivity additive. In Figure 7B, OAm-GO/CZTS has a Tafel slope of 64 mV/dec. The value is greater than the Pt 36 mV/dec, but significantly lower than OAm-115 GO mV/dec, pure CZTS 85 mV/dec, OAm-CZTS 83mV/dec, and GO/CZTS 81mV/dec. In Figure 7C shows the impedance spectra of OAm-GO/CZTS. The charge transfer resistance (*R*_*ct*_) at the electrode and electrolyte contact causes the observed semi-circle. According to the impedance fitting, the OAm-GO/CZTS has a reduced charge transfer resistance (*R*_*ct*_) of 5Ω, compared to the GO/CZTS (23Ω), OAm-CZTS (37Ω), OAm-GO (50Ω), and GO (78Ω). Lowering the Rct value of the OAm-GO/CZTS improves the electron transfer processes, which helps to improve HER. The catalytic stability of the OAm-GO/CZTS in a 0.5M H_2_SO_4_ solution was proven, indicating that it might be used in practice. After 1000th CV cycles, the OAm-GO/CZTS polarization curves in Figure 7D demonstrate a minor loss of HER catalytic activity. The chronopotentiometry curve in the inset of Figure 7D further indicates that the OAm-GO/CZTS could produce a consistent current density at an overpotential of 47 mV for 1,000 min with no performance loss. These findings show that the OAm-GO/CZTS is extremely durable in the HER environment (Kush et al., 2015).

### 5.7 Heterostructured CZTS based electrocatalysts for HER

**FIGURE 8:**
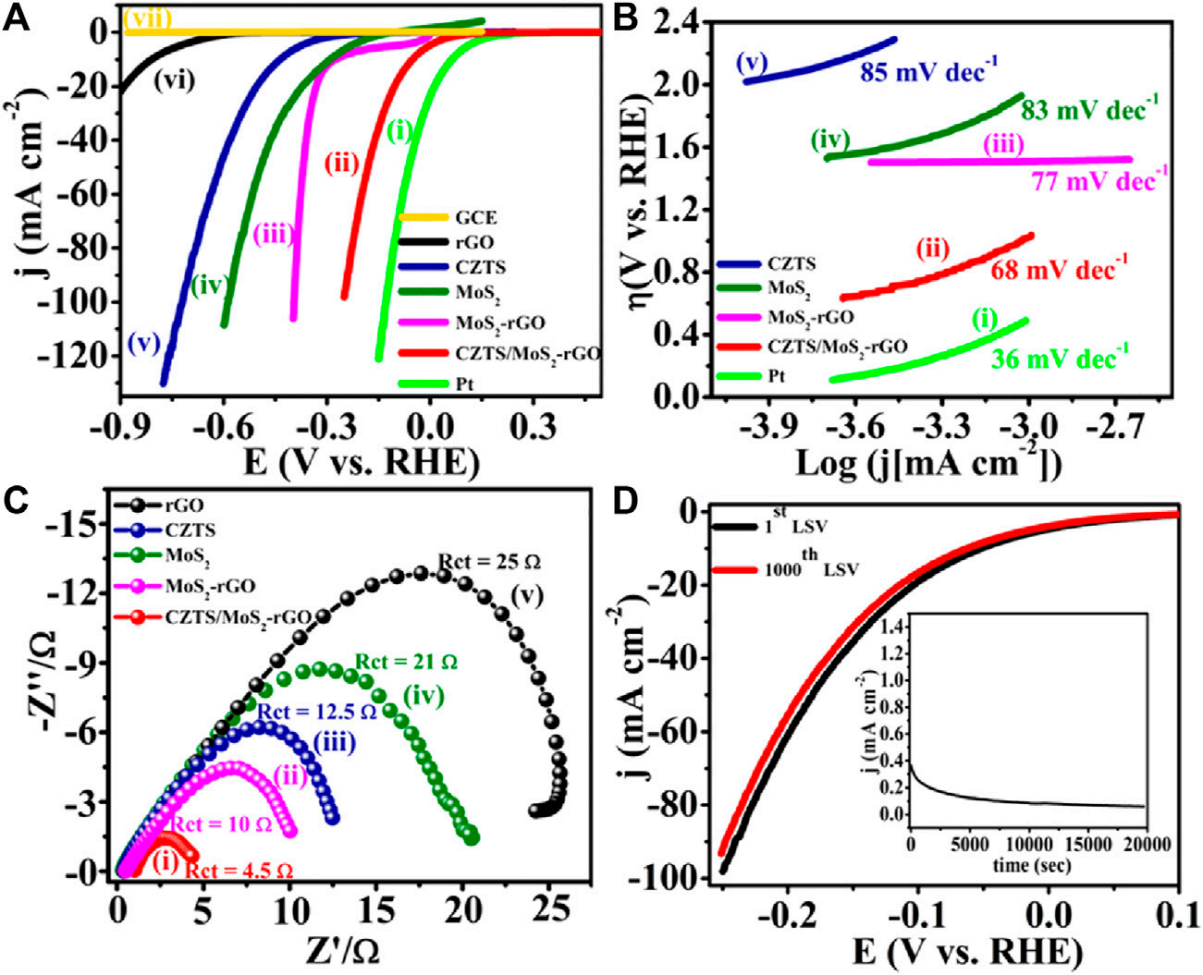
**(A)** Superimposed HER polarization curves showing the highest catalytic activity of CZTS/MoS_2_-rGO; **(B)** corresponding to the Tafel plot; **(C)** Nyquist plot; **(D)** The durability test of CZTS/MoS_2_-rGO using SCE and Pt wire as reference and counter electrodes in 0.5 M H_2_SO_4_ at 50 mV/s.

The design and development of inexpensive, highly efficient electrocatalysts for hydrogen production is the basis of several potential sustainable energy solutions. For enhanced hydrogen generation procedures (HER), Renuka et al. developed a straightforward sonochemical method for a novel heterostructured electrocatalyst composed of MoS_2_-rGO and Cu_2_ZnSnS_4_ (CZTS) NPs (CZTS/MoS_2_-rGO) which showed in Figure 8. Figure 8A shows the polarization curves of several electrocatalytic systems, such as bare GCE, Pt, rGO, pristine MoS_2_, pure CZTS, and MoS_2_-rGO. The polarization curves demonstrate that the CZTS/MoS_2_-rGO heterostructure, with onset overpotentials of 50 and 102 mV to enable current densities of 10 mA cm^−2^ and 20 mA cm^−2^, has exceptional electrocatalytic performance towards HER activity. Under the specified circumstances, this improvement is greater than that of previously published CZTS (300 mV), Co-CZTS (298 mV), and MoS_2_ (180 mV) based HER catalytic systems. The synergistic effect of MoS_2_-rGO and CZTS as co-catalysts, which provide a conducting network for electron transport and increase the abundance of exposed edges, which act as active catalytic sites for the HER, is thought to be responsible for the enhanced electrocatalytic activity of the CZTS/MoS_2_-rGO heterostructure. Using graphite as a counter electrode, the LSV polarization curve revealed a negligible shift in the overpotential.

Figure 8B shows the Tafel slope of around 36, 83, 85, and 77 mV dec^−1^ is seen for Pt, MoS_2_, CZTS, and MoS_2_-rGO, respectively, in the Tafel plots for rGO, pure MoS_2_, CZTS, MoS_2_-rGO, and CZTS/MoS_2_-rGO. In contrast to these materials, CZTS/MoS_2_-rGO has a comparatively lower Tafel slope (68 mV dec^−1^) and stronger catalytic activity towards HER. The CZTS/MoS_2_-rGO electrocatalyst continues to exhibit a comparatively low Tafel value when compared to the literature. There are three possible reaction steps which have been suggested for the HER, such as, in the acidic medium a primary discharge step (Volmer reaction), electrochemical desorption step (Heyrovsky reaction) and recombination step (Tafel reaction). For each of the three stages, the theoretical Tafel values are 40, 30, and 120 mV per decade, respectively. Determining and interpreting the Tafel slope is crucial to understanding the basic processes involved. The current work's Tafel slope observation of the CZTS/MoS_2_-rGO catalyst (68 mV dec^−1^) indicates that the rate-determining processes are likely to be electrochemical desorption and primary discharge. To sum up, the research offers a significant understanding of the polarization curves of different electrocatalytic systems and emphasizes their potential to enhance HER catalysis performance. The results also offer a foundation for additional study and advancement in the area of electrocatalysis. With an exchange current density (j0) of 9.62 mA/cm^2^, which suggests quicker electrochemical charge transfer via the catalyst-solution interface, the study shows that CZTS/MoS_2_-rGO is an effective electrocatalyst for H_2_ evolution. Compared to numerous documented non-noble metal HER catalysts, this value is greater. The results of electrochemical impedance spectroscopy (EIS) indicate that the charge transfer-based parameters are based on quicker charge transfer kinetics showed in Figure 8C. The Rct value of CZTS/MoS_2_-rGO is 4.5 Ω. The Rct values of the other electrode materials, rGO, MoS_2_, CZTS, and MoS_2_-rGO, are 25, 21, 12.5, and 10 Ω, in that order. The electrocatalyst's stability performance was tested for 1000 CV cycles at a scan rate of 50 mV s^−1^ showed in Figure 8D. Little loss is visible in the LSV polarization curves of the first and 1000th LSVs following the last cycle. The i-t chronoamperometric test for CZTS/MoS_2_-rGO at an overpotential of 54 mV, slightly higher than the overpotential, suggested that the activity would stay the same even if the HER activity of the compound was determined to be as good as the same. By applying Jaramillo's approach, the turnover frequency (TOF) of H2 molecules evolved per second. With a TOF value of 0.354867 s^−1^ at η = 250 mV, CZTS/MoS_2_-rGO exhibited the greatest intrinsic activity of any active site. The electrochemical double-layer capacitance of the catalytic surface was used to determine the electrochemically active surface area (ECSA) for each system. The CZTS/MoS_2_-rGO electrocatalytic system's computed ECSA is 20.42 cm^2^. The MoS_2_-rGO co-catalyst, which is supported by the surface of CZTS, provides the exceptional electrochemical performance of CZTS/MoS_2_-rGO. While MoS_2_ carries electrons towards semiconducting CZTS nanoparticles, electrically conductive rGO sheets help carry electrons to the conducting MoS_2_ catalyst. The highly conductive and active catalyst exhibits quick electron kinetics, as seen by the reduced semicircle diameter of (Rct = 4.5 Ω), which offers good charge-transfer capabilities.

### 5.8 Metal doped CZTS based OER (water oxidation)

Renuka et al. demonstrated that the sonochemical method were used for the synthesis of nickel and iron (M = Ni, Fe) doped Cu_2_ZnSnS_4_ (CZTS) as a nanoelectrode for better electrocatalytic water splitting performance. Transmission electron microscopy (TEM), X-ray diffraction (XRD), Raman, and X-ray photoelectron (XP) spectroscopy experiments were used to further characterize the as-prepared electrode materials. Significantly, in 0.5 M H_2_SO_4_ and 1 M KOH electrolyte solutions, Ni-doped CZTS electrocatalysts have low overpotentials of approximately 214 and 400 mV vs. RHE for HER, respectively, and 1.29 V vs. RHE for OER in 1 M KOH at a current density of 10 mA/cm^2^. Small Tafel slopes and durability test, up to 500 min for water splitting, show that Ni-doped CZTS is an excellent bifunctional electrocatalyst with high activity and exceptional stability. Furthermore, the Fe-doped CZTS electrocatalyst has a poor response, with an overpotential of 300 mV in 0.5 M H_2_SO_4_ and 445 mV in 1.0 M KOH towards HER and 1.54 V vs. RHE for the OER in 1 M KOH reaching at 10 mA/cm^2^. This high-efficiency bifunctional electrocatalyst can address the current energy scarcity.

**FIGURE 9:**
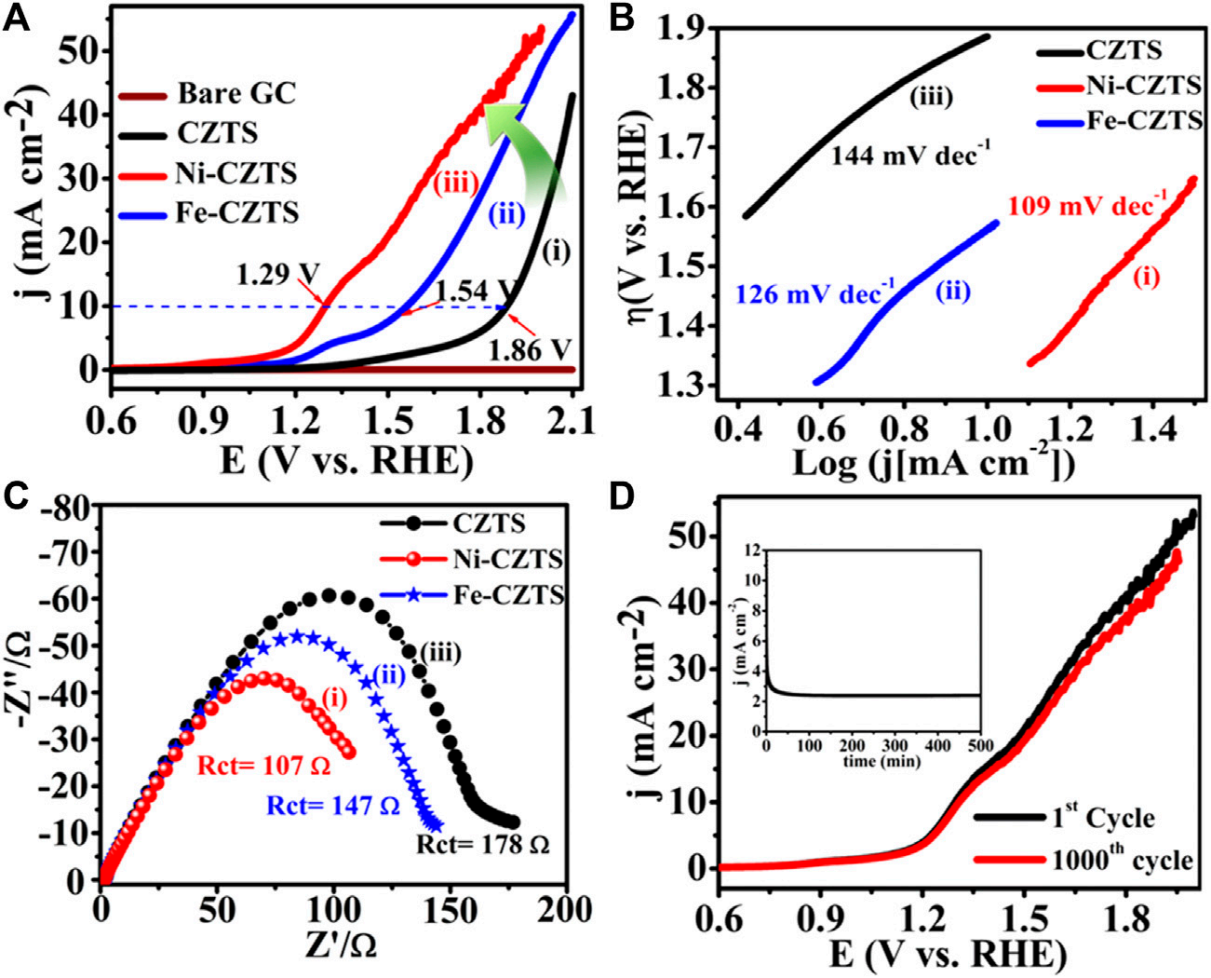
**(A)** OER LSV curves, **(B)** corresponding to the Tafel plot, **(C)** Nyquist plot, **(D)** The durability test of Ni-CZTS CZTS using SCE and Pt as reference and counter electrode is in 1.0 M KOH.

Doping is one of the most effective ways to improve electrocatalytic activity, which is why this study focused on Ni and Fe doped CZTS nanoparticles. The Ni-doped CZTS electrocatalyst has a low overpotential of around 1.29 V vs. RHE for the OER in 1 M KOH electrolyte solution at 10 mA/cm^2^current density, as shown in Figure 9A. Furthermore, the Fe-doped CZTS electrocatalyst has a weak response, with an overpotential of 1.54 V in 1 M KOH reaching 10 mA/cm^2^. As illustrated in Figure 9B, the matching Tafel slopes were obtained to analyze OER kinetics. When compared to CZTS (144 mV/dec) and Fe-CZTS, the resulting Tafel slope of Ni-CZTS is 109 mV/dec, which is the lowest (126 mV/dec). These findings demonstrated that Ni-CZTS outperforms CZTS and Fe-CZTS catalysts, as well as the previously published OER-based catalysts, in terms of electrocatalytic characteristics. Spectroscopy of electrochemical impedance (EIS). The semicircular diameter of the Ni-CZTS (*R*_*ct*_ = 107) in the Nyquist plot is lower than that of the CZTS (*R*_*ct*_ = 178) and Fe-CZTS (*R*_*ct*_ = 147), as shown in Figure 9C. The Ni-CZTS electrode has a considerably lower charge transfer resistance than the Fe-CZTS electrode, suggesting a lot quicker electron transfer and consequently greater OER electrocatalytic activity, according to the EIS. The increased electrical conductivity and smaller nanoparticle size (Figure 9) enhance charge and mass transfer, as well as rapid diffusion and reactivity at the electrolyte-electrode interface. Electrocatalyst durability is an important consideration for practical applications. As a result, the electrocatalyst’s polarization curve after 1000th cycles show high electrocatalytic stability for the OER.

In Figure 9D, the electrocatalyst is substantially comparable to the original, with just little degradation. Furthermore, the long-term stability for 500 min of Ni-CZTS was carried by using chronopotentiometry test, as shown in the inset of Figure 9D, which validates the durability of Ni-doped with little anodic current loss in alkaline electrolyte. These findings show that the Ni-CZTS catalyst outperforms conventional OER electrocatalysts in terms of activity (Digraskar et al., 2019a). Significantly, as a consequence of OER activity, the electrocatalytic performances of Ni and Fe doped CZTS NPs have been observed as Ni-CZTS > Fe-CZTS > CZTS, which is compatible with the morphological features.

### 5.9 Carbon nanostructured composites of CZTS for OER

**FIGURE 10:**
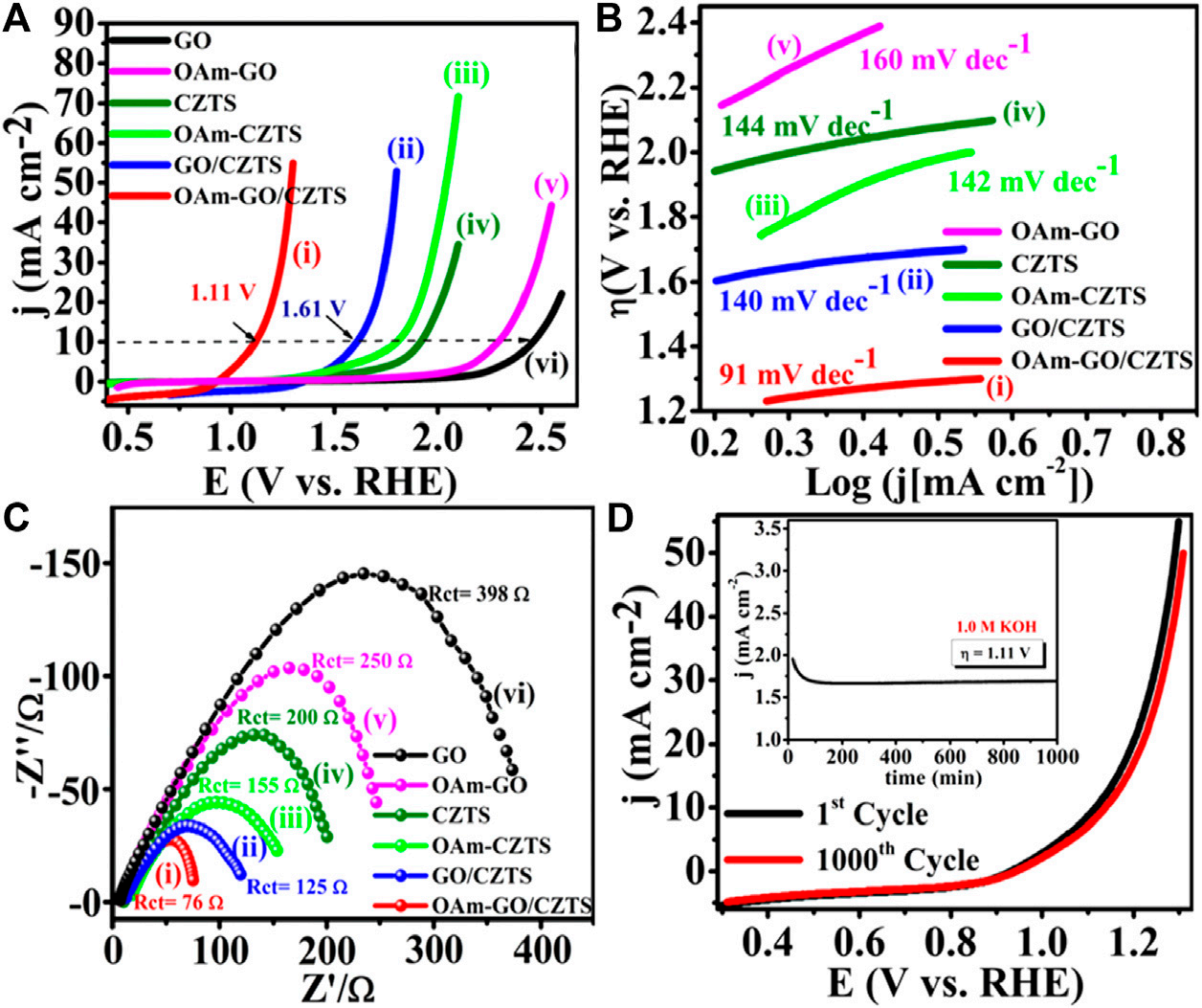
**(A)** Superimposed OER polarization curves showing the highest catalytic activity of OAm-GO/CZTS; **(B)** corresponding to the Tafel plot, **(C)** Nyquist plot, **(D)** The durability test of OAm-GO/CZTS (inset of (d)) shows i-t test for 1,000 min in 1.0 M KOH.

Employing non-noble metal-based bifunctional electrocatalysts for the overall water splitting reaction emerges as a highly promising strategy in addressing concerns related to greenhouse gas emissions and energy sustainability. An oleylamine functionalized graphene oxide/Cu_2_ZnSnS_4_ composite (OAm-GO/CZTS) was produced and studied in this work as an advanced bifunctional electrocatalyst for OER which demonstrated in Figure 10. The produced catalysts have notable OER performance, as determined by LSV in a basic electrolyte solution (1.0 M KOH) at a scan rate of 50 mV s^−1^ at room temperature, as demonstrated by electrocatalytic experiments showed in Figure 10A. The Tafel slope of OAm-GO/CZTS is 91 mV dec^−1^ in Figure 10B. Remarkably, OAm-GO/CZTS shows a low overpotential of η = 1.36 V at 10 mA cm^−2^, and the observed overpotential for OER is among the lowest that has been observed to date. The electrocatalysts OAm-GO (160 mV dec^−1^), CZTS (144 mV dec^−1^), OAm-CZTS (142 mV dec^−1^), and GO/CZTS for (140 mV dec^−1^) have the lowest values of this. The electrocatalytic characteristics of OAm-GO/CZTS are found to be superior to those of other catalysts, including recently published OER-based catalysts, as demonstrated by these data. Moreover, Figure 10C displays the semicircular diameter. Because of its lower electron transfer resistance in alkaline solution, the OAm-GO/CZTS (*R_ct_* = 76 Ω) is significantly inferior than GO/CZTS (*R_ct_* = 125 Ω), OAm-CZTS (*R_ct_* = 155 Ω), CZTS (*R_ct_* = 200 Ω), OAm-GO (*R_ct_* = 150 Ω), and GO (*R_ct_* = 398 Ω). As seen in Figure 10D, the OAm-GO/CZTS has exceptional endurance in the alkaline electrolyte. The OAm-GO/CZTS catalyst maintains its original look and polarization curve even after 1000th continuous cycles. Compared to other OER electrocatalysts, this outcome is much more fantastic. The inset of Figure 10D displays the results of the chronoamperometric (CA) test for 1000th minutes, which demonstrates the exceptional stability performance of OAm-GO/CZTS with a very little anodic current loss in the alkaline electrolyte. In other words, the following factors are primarily responsible for the suggested OAm-GO/CZTS electrocatalyst's higher OER performance: Firstly, the higher catalytic activity is mostly attributed to amine functionalization. The amine group's lower |ΔGH*| value boosts the electron transferability of GO, which is advantageous for the water-splitting process. Second, the massive specific surface area of the OAm-GO/CZTS electrocatalyst facilitates the bulk transport of ions from GO into the electrolyte, improving the catalytic performance of HER. Third, when graphene has been oxidized and OAm functionalized, it exhibits exceptional OER performance. This is because the graphene surface has a large number of amino groups and oxygen atoms, which increase its catalytic activity. Fourth, quick electron transport from the very resistive CZTS NPs to the electrodes was made possible by electronic coupling to the basic GO consistent conducting system. Therefore, the greater electrocatalytic activity of the OAm-GO/CZTS may have been caused by the quick charge transfer during the electrocatalytic process, which remained constant with a large exposed active surface area. Fifth, quick dispersion and reaction at the electrolyte-electrode interface are facilitated by the synergistic interaction between conducting GO composite and transition-metal chalcogenide (CZTS), which speeds up charge transfer. OAm-GO/CZTS is a competent and stable bifunctional overall water-splitting electrocatalyst, according to the justification provided above. In summary, the OAm-GO co-catalyst supported by the CZTS surface provides the electrochemical performance of OAm-GO/CZTS.

## 6 Conclusion and future prospectus

A summing up of all the foremost conclusions of the present review with reverence to the production of CZTS NPs and their surface amendment, heterostructure configuration along with different carbon nanostructured and related materials followed by their tunable electrocatalytic studies towards water splitting reaction, and their structure-property relationships are discussed. Single of the key studies is the synthesis of tiny CZTS nanoparticles with tunable reactivity and their presumable mechanism of formation by the sonochemical method. Still, despite the fact that CZTS is a low-cost catalytic system, it typically endures from poor HER behaviour. Pleasing these factors into the report, carbon materials such as graphene, carbon nanotube, C_60_ are ultimate ropes to get better electrocatalytic activity due to their distinctive physicochemical properties. A straightforward way of amine functionalization of GO/CZTS composite has been discussed with superior overall water splitting performance. This could be attained owing to the electron donating effect of amine groups and sympathetic electrical conductivity of graphene. In totaling, doping is one of the best strategies to promote catalytic performance. There are several doped electrocatalytic systems, for instance, non-noble metal Fe, Co., Ni-doped with CZTS enhances the catalytic activity than pure CZTS. Moreover, heterostructure consisting of CZTS nanoparticles attached to a MoS_2_- reduced graphene oxide (rGO) hybrid. via. a facile single-step sonochemical technique, it originates that compared with pure CZTS and MoS_2_, the CZTS/MoS_2_-rGO heterostructure have the enhanced activity for HER, confirm that the introduction of carbon materials and MoS_2_ (MoS_2_-rGO hybrids) could get better the performances of CZTS-based electrocatalysts for HER. Growing demand for energy and concerns to develop efficient alternative renewable energy sources is the need of the time, where efficient hydrogen generation as alternative energy is promising. With this motivation, the main objective of this review work is to further demonstrate an efficient and cost-effective electrocatalysts for hydrogen generation reactions, the tremendous progress recently achieved on CZTS have demonstrated the potential of fabricating high-performance and cost-effective material with low environmental pollution. Both vacuum-based and non-vacuum-based methods have been successfully explored to fabricate CZTS. Among vacuum-based methods, evaporation and sputtering are appropriate deposition techniques in terms of efficiencies. Non-vacuum-based technique including nanoparticle based and sol-gel methods, the one step sonochemical method are promising because of simplicity. To further improve the performance of CZTS wherein, metal nanostructures anchored on carbon-based materials (enhancing surface area/activity) demonstrate extraordinary electrocatalytic performance in water splitting reactions. This system together with the possibility of modulating their co-operative interactions and tuning their characteristic properties, serve to be one of the remarkable features of this energy generation device.

Electrocatalysts based on CZTS have a bright future in the electrochemical water splitting process. The following are some important topics to watch and future developments:

### 6.1 Enhanced activity and stability

By enhancing the materials’ composition, structure, and surface characteristics, scientists are attempting to increase the catalytic activity and stability of CZTS-based materials. This involves creating brand-new synthetic methods and improving performance by adding dopants or co-catalysts.

### 6.2 Effective charge transfer

It is imperative to improve the effectiveness of charge transfer mechanisms at the electrode-electrolyte interface. Charge transfer kinetics and overall catalytic performance can be enhanced by techniques including interface engineering, heterostructure creation, and surface modification.

### 6.3 Cost-effectiveness and scalability

Upcoming studies seek to provide synthesis techniques for CZTS-based electrocatalysts that are both affordable and scalable. This may entail the use of widely available, reasonably priced precursors in addition to straightforward manufacturing methods appropriate for mass production.

### 6.4 Durability and lifespan

For practical applications, CZTS-based electrocatalysts’ durability and lifespan must be improved. The goal of research is to create materials that are very stable in the face of challenging electrochemical processes including electrodeposition and corrosion.

### 6.5 Integrating with renewable energy sources

To construct integrated systems for sustainable hydrogen generation, CZTS-based electrocatalysts may be combined with renewable energy sources like solar panels. This could aid in the advancement of renewable and clean energy technology.

### 6.6 Gaining more insight into reaction processes

It is imperative to gain further insight into the processes behind the electrochemical water splitting process on CZTS-based electrocatalysts. This information can direct the development of catalysts that are more stable and effective.

All things considered, there is a bright future ahead of CZTS-based electrocatalysts for the electrochemical water splitting process. Research is now being done to improve catalytic performance, durability, affordability, and integration with renewable energy sources. These developments may have a major impact on the creation of sustainable and effective hydrogen generation methods.
